# Engineered protein corona sustains stealth functionality of nanocarriers in plasma

**DOI:** 10.1186/s12951-025-03565-x

**Published:** 2025-07-14

**Authors:** Xueqing Zhang, Shutian Si, Ingo Lieberwirth, Katharina Landfester, Volker Mailänder

**Affiliations:** 1 Department of Dermatology, University Medical Center Mainz, Langenbeckstraße 1, 55131 Mainz, Germany; 2 Max Planck Institute for Polymer Research, Ackermannweg 10, 55128 Mainz, Germany

**Keywords:** Nanocarrier, Protein corona, Preadsorption, Stealth, Human plasma, Cell uptake, Protein denaturation

## Abstract

Pre-adsorption of selective proteins is instrumental in forming functionally important protein coronas that allow for achieving long circulation in blood stream. This is an important prerequisite for subsequent specific uptake into target cells. Despite a wide range of studies aimed at identifying these effective pre-adsorbed proteins, the separate experiments and varying conditions carried out in human and murine systems, respectively, have left a gap in our comprehensive understanding of how these proteins help to maintain the stability of dysopsonin character at both the intra- and inter-species level. To address this, we systematically experimented with human- and murine-derived stealth proteins, clusterin and apolipoprotein A1, to engineer protein coronas of nanoparticles. We then exposed the pre-coated nanoparticles to human and murine blood plasma and evaluated the stability of the stealth protein corona and the stealth properties against macrophages. Our proteomics data elucidate that the further recruitment in the amount of the two stealth proteins before and after pre-adsorption accounts for the stable targeting property of nanoparticles by enabling them to evade macrophage phagocytosis (stealth effects). However, significant discrepancies were observed between these interspecies experiments, which is largely attributed to diverse protein corona compositions. These observations shed lights on the guidance to overcome conversion barriers from murine experiments to human clinical trials.

**Supplementary Information:**

The online version contains supplementary material available at 10.1186/s12951-025-03565-x.

## Introduction

The presence of stealth nanocarriers is important to prevent unspecific uptake of nanocarriers by macrophages or other cells in the blood plasma. Unmodified nanoparticles are predominantly taken up by phagocytes and therefore mostly end up in the liver and spleen, where many phagocytically active cells reside or locate to [1]. This has been attributed to the presence of adsorbed proteins when these nanoparticles come into contact with protein-containing body fluids, mostly blood plasma proteins [2–4]. In order to avoid non-specific uptake, coating of nanoparticles with stealth polymers has been successfully implemented [5]. Specifically, this can be obtained indirectly by functionalization of stealth polymers on the particle surface or, much more simply, directly by adsorption of proteins with stealth properties. In the latter case, the stability of the absorbed protein layer in the plasma is crucial and has not been studied in detail. With this regard, we have now investigated the stability of an engineered protein corona of nanocarriers in blood plasma. Engineering a protein corona for nanocarriers is essential to achieve a stealth effect. We and others have shown that the stealth properties of e.g. polyethylene glycol (PEG) are not conferred by completely preventing the adsorption of proteins, as has long been suggested, but by adsorbing specific proteins [6–9]. Namely clusterin (also known as apolipoprotein J) and apolipoprotein A1 (ApoA1) have been found on PEGylated nanoparticle surfaces. These “stealthy proteins” are therefore identified as “dysopsonins” [10]. Both of them play an important role in lipid metabolism in the body, facilitating to transport cholesterol to the liver. Beyond lipid transport, they also regulate inflammatory immune response by interacting with the complement system [11]. Particularly ApoA1, as high-density lipoprotein (HDL), has been reported to associate with immune cell receptors, thereby affecting downstream signaling pathways [12, 13]. Since PEG has also been shown to be immunogenic [14, 15] we proposed to us ne proteins directly as surface functionalization since they are completely endogenous molecules. They are also present in high amounts in plasma and therefore immunological reactions against them are not expected to occur [16–18].

However, the test results of animal models are not always applicable to clinical trials [19]. Especially when particles are exposed to different protein sources, potential agglomeration and aggregation issues can bring uncertainty to the biodistribution [20, 21]. Therefore, the exploration across protein sources and phagocytic behavior of interspecies cell lines is of great significance.

The distinct composition of protein corona arises from external protein sources as well as the surface properties of nanoparticles themselves. These observations shed lights on the necessity of caution when transferring the knowledge gained from murine experiments to human clinical trials. Fortunately, a substantial number of strategies, such as regulating the polymer hydrophilicity [22] or employing carbohydrate (HES, Dextran or Glucose) coatings [23]have been developed to selectively control protein adsorption. These studies demonstrate the potential to overcome the barriers in translating therapeutics from mouse models to human applications. The similarity between all these stealth nanocarriers is that clusterin or apolipoprotein A1 was observed to dominate their corona pattern, which directly inspired us to choose these two proteins as a single precoating for our study.

Earlier studies established that apolipoproteins have affinity for the synthetic moieties of polystyrene NPs [24, 25]and several studies have found that apolipoproteins bind to poly(ethylene glycol) (PEG) functionalized nanocarriers [26]particularly for lipid-based nanoparticles [27, 28]. As coating with single proteins such as clusterin and ApoA1 is easily achieved by simple co-incubation and adsorption of the proteins on the nanoparticle surface, we set out to investigate how stable such a non-covalent surface modification would be. The main objective of this study was to determine if precoating with a single protein could withstand further incubation in whole plasma. This has not been thoroughly investigated, but is of great importance for the development of this strategy of protein precoating as a stealth treatment.

**Fig. 1:**
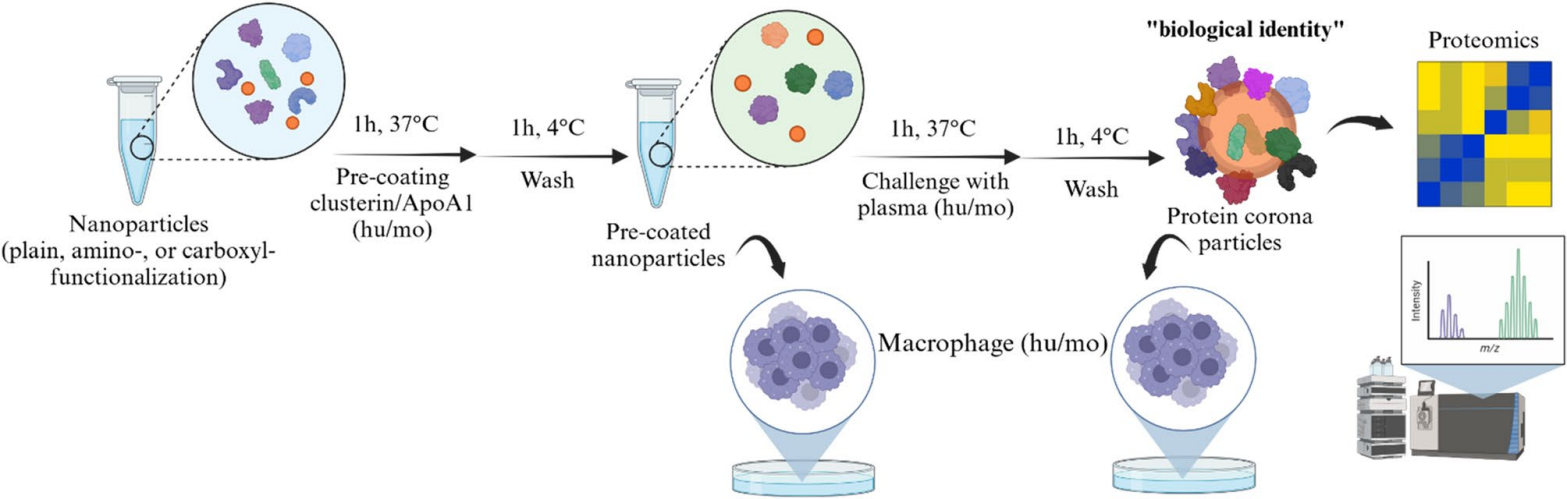
Schematic overview: First, we pre-coated three different polystyrene nanoparticles (plain, amino-functionalized and carboxyl-functionalized) with ApoA1 or clusterin from human and mouse sources. We then challenged these pre-coated particles with human and mouse citrated plasma. We then checked the protein corona fingerprints using LCMS. Finally, we investigated potential differences in the uptake of these modified nanoparticles by human and murine macrophages

To follow up on our previous work and that of others on the influence of the protein corona on uptake and stealth effect, we used the same chemically modified polystyrene (PS) particles (stabilized with Lutensol AT50 as PEG analog) as before [20, 29, 30] with unmodified (plain), amino- or carboxyl-functionalized surfaces by co-polymerization with co-monomers. These are then localized on the nanoparticles’ surface [31, 32]. Subsequently, we adsorbed stealth proteins: human (hu) and mouse (mo) apolipoprotein A1 (ApoA1) and clusterin (clusterin) onto the nanoparticles as a pre-coating and then challenged the pre-coating by incubation in whole plasma (all mouse and human). As a secondary question, we investigated whether proteins from different species have the same effect on challenge in plasma from these different species and whether the biological stealth effect is preserved between species. Label-free liquid chromatography coupled to mass spectrometry (LC-MS) was used to determine how much of the precoating was retained after plasma challenge (Fig. 1).

## Results and discussion

Since the pre-coating altered the surface property of the particles, this could be observed by protein quantification (Pierce assay). In an attempt to titer the amount of individual proteins, we used two concentrations of clusterin and ApoA1 for precoating: a low concentration of 30 µg and a high concentration of 120 µg per 0.05 square meter of particle surface area to examine the stealth efficacy of the engineered protein corona across species protein sources and to investigate potential differences in cellular uptake by human and murine macrophages.

**Fig. 2:**
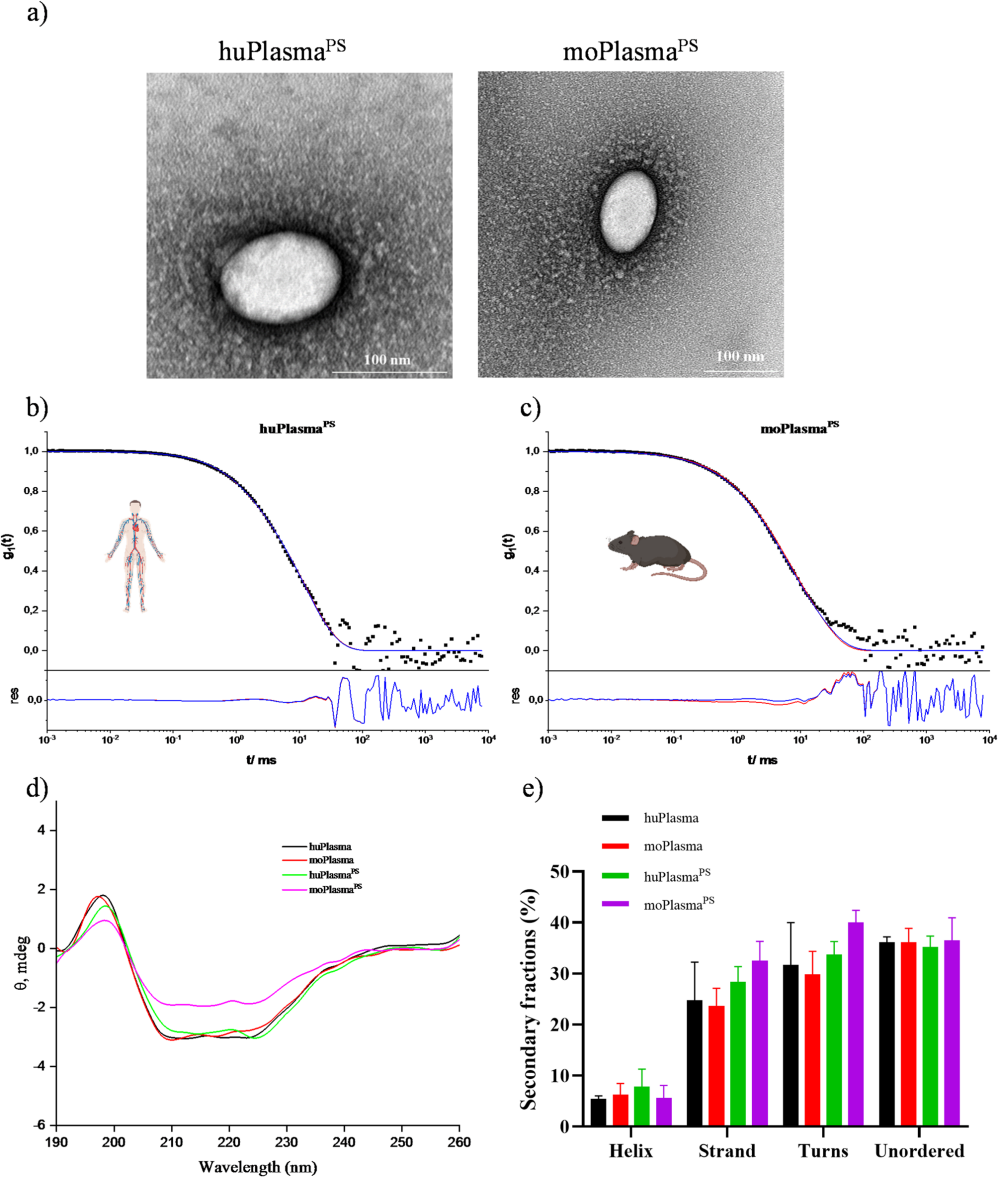
**(a)** TEM micrographs of unfunctionalized PS NPs incubated with human or mouse plasma, centrifuged and washed three times. The protein corona surrounding the NPs is visualized. Samples were stained with 4% uranyl acetate for better contrast. Scale bar: 100 nm. Determination of the aggregation tendency of unfunctionalized PS NPs in concentrated **(b)** human or **(c)** murine plasma sources by dynamic light scattering (DLS). (Top) Exemplary autocorrelation functions (ACFs) at a scattering angle of 30° of nanoparticles in each plasma source together with the corresponding fits with (blue line) and without (red line) an additional aggregation term. (Bottom) Residuals resulting from the difference between the data points and both fits. **(d)** CD spectra of native plasma and after protein corona formation on unfunctionalized PS NPs. Particles were treated with huPlasma or moPlasma for 1 h, 37 ℃. Then they were washed once to remove unbound proteins. **(e)** Secondary structure fraction was calculated by CDPro software (SELCON3, CONTINLL, and CDSSTR)

To fully characterize the three different polystyrene nanoparticles (plain, amino-functionalized, and carboxyl-functionalized) with or without pre-coating treatment and before and after plasma challenge with different sources, we performed transmission electron microscopy (TEM) (Fig. 2a), dynamic light scattering (DLS), ζ-potential (Figure S1), and circular dichroism (CD) spectrum measurements (Fig. 2d-e).

As can be seen in Fig. 2a, it is clear that the protein corona around the particles is structured in a loose network rather than in a densely packed manner. In addition, we can see the protein corona grafted to the particle surface in situ with Cryo-TEM images (Figure S2) when the whole plasma is used, whereas we were not able to detect protein coats in TEM when using single proteins such as huApoA1 (Figure S2). It is easier to see the protein corona around NPs after plasma exposure, but we cannot clearly detect it with only single pre-coating treatment, which is simply due to the low amounts of adsorbed proteins. Using dynamic light scattering (DLS), we were able to compare the size changes with and without protein coatings. For unfunctionalized polystyrene (PS) nanoparticles (Figure S1a), the size increased to approximately 10 nm for precoating, 30 nm for human plasma challenge, and 20 nm after mouse plasma challenge treatments. The results for amino-functionalized polystyrene nanoparticles (PS-NH2) are similar (Figure S1c): the size increased to about 15 nm for pre-coating, 30 nm for human plasma challenge and 20 nm after mouse plasma challenge treatments, respectively. Furthermore, the corresponding polydispersity indices (PDI) for PS- and PS-NH2-based NPs are all below 0.3 (Figure S1 Table), indicating a relatively narrow size distribution. However, carboxyl-functionalized polystyrene nanoparticles (PS-COOH) showed heterogeneity after challenge with mouse plasma (Figure S1e): pre-coating with moApoA1 or moClusterin always tends to increase the size. The overall size can increase to 300 ~ 700 nm with a broad size distribution (PDI > 0.5), much larger than pristine PS-COOH NPs (diameter: 145 nm, PDI: 0.177). While PS-COOH NPs remain stable after incubation with human plasma, showing a size increase of 50 ~ 100 nm. Since aggregation and agglomeration can cause an increase in particle size20-26, multi-angle dynamic light scattering allows us to evaluate the aggregation behavior of particles in plasma without applying any washing steps [33]. As shown in Fig. 2b-c and S3, we observed negligible aggregation in the human plasma system, but slight aggregation for PS-COOH when incubated in mouse plasma (Figure S3d). This aggregation will affect interactions with cells and biodistribution in vivo [34]. Protein coating not only altered the size and stability of the particles, but also the surface charge characteristics (Figure S1b, d, f). The zeta potential measurements showed that the values of uncoated PS-NPs were − 17 mV, PS-NH2 were − 14 mV and PS-COOH were − 33 mV. Compared to human plasma (PS: -20 mV, PS-NH2: -24 mV, and PS-COOH: -25 mV) and precoating (PS: -23 mV, PS-NH2: -24 mV, and PS-COOH: -30 mV) treatments, mouse plasma coating resulted in a more negative surface charge of ~ -27 mV in most cases. The composition of the adsorbed proteins as well as the secondary structure of the proteins affect the type of receptors on cells that interact with protein-coated NPs, such as albumin receptors, scavenger receptors etc [35]. As shown in Fig. 2d-e, circular dichroism (CD) spectra suggested that there was no detectable denaturation after the absorption process, as no shift in secondary structure was observed before and after protein corona formation by circular dichroism [36–38].

**Fig. 3:**
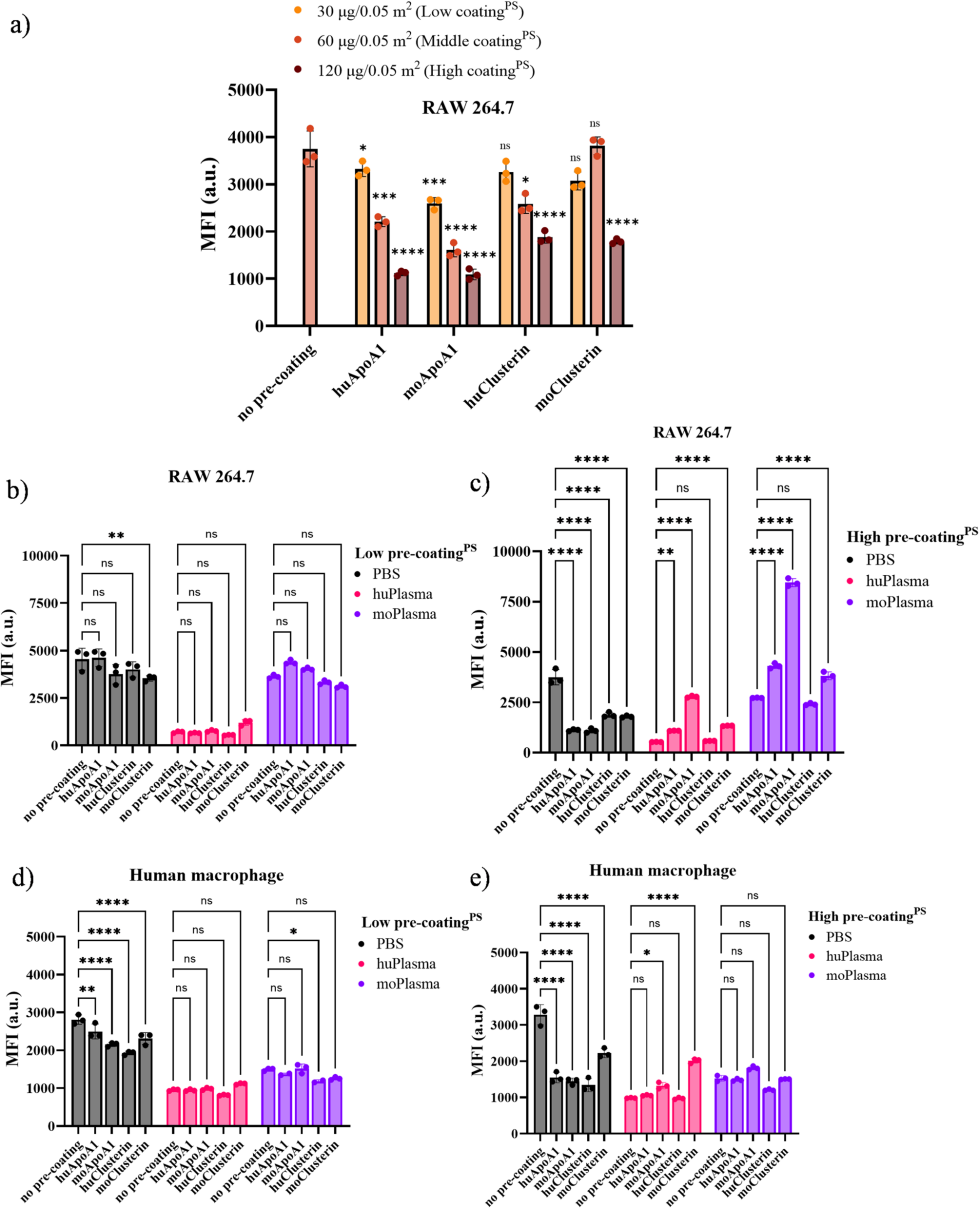
Flow cytometric analysis: Macrophages were incubated with 40 µg/mL unfunctionalized PS NPs for 2 h. **a)** Prior to RAW 264.7 cellular uptake studies, pristine and ApoA1 or clusterin (low: 30 µg, middle: 60 µg, high: 120 µg, per 0.05 m2 surface area) pre-coated PS NPs were incubated for 1 h, 37℃. Wash once to remove unbound proteins. Before b-c) RAW 264.7 and **d-e)** human macrophage cellular uptake studies, pristine and ApoA1 or clusterin **b**,** d)** low: 30 µg, **c**,** e)** high: 120 µg, per 0.05 m2 surface area pre-coated PS NPs for 1 h, 37℃. Wash once to remove unbound proteins. Then challenge the precoated particles with human plasma or mouse plasma for 1 h, 37 ℃. Wash again to remove unbound proteins. Values are expressed as mean ± standard deviation (SD, *n* = 3)

**Fig. 4:**
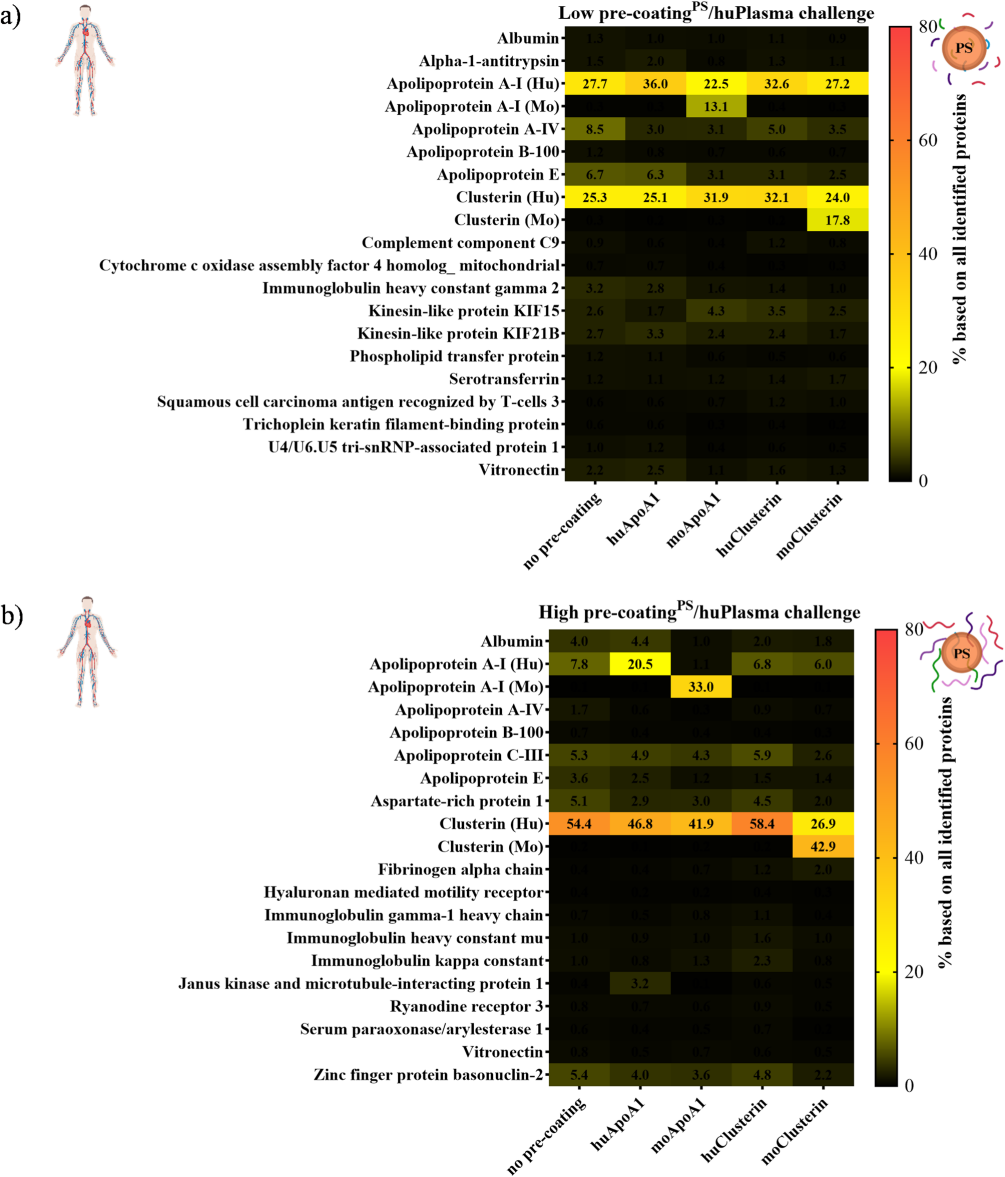
LC-MS analysis of corona composition: pristine and ApoA1 or clusterin **(a)** low: 30 µg, **(b)** high: 120 µg, per 0.05 m2 surface area of precoated PS NPs, followed by challenge with human plasma

**Fig. 5:**
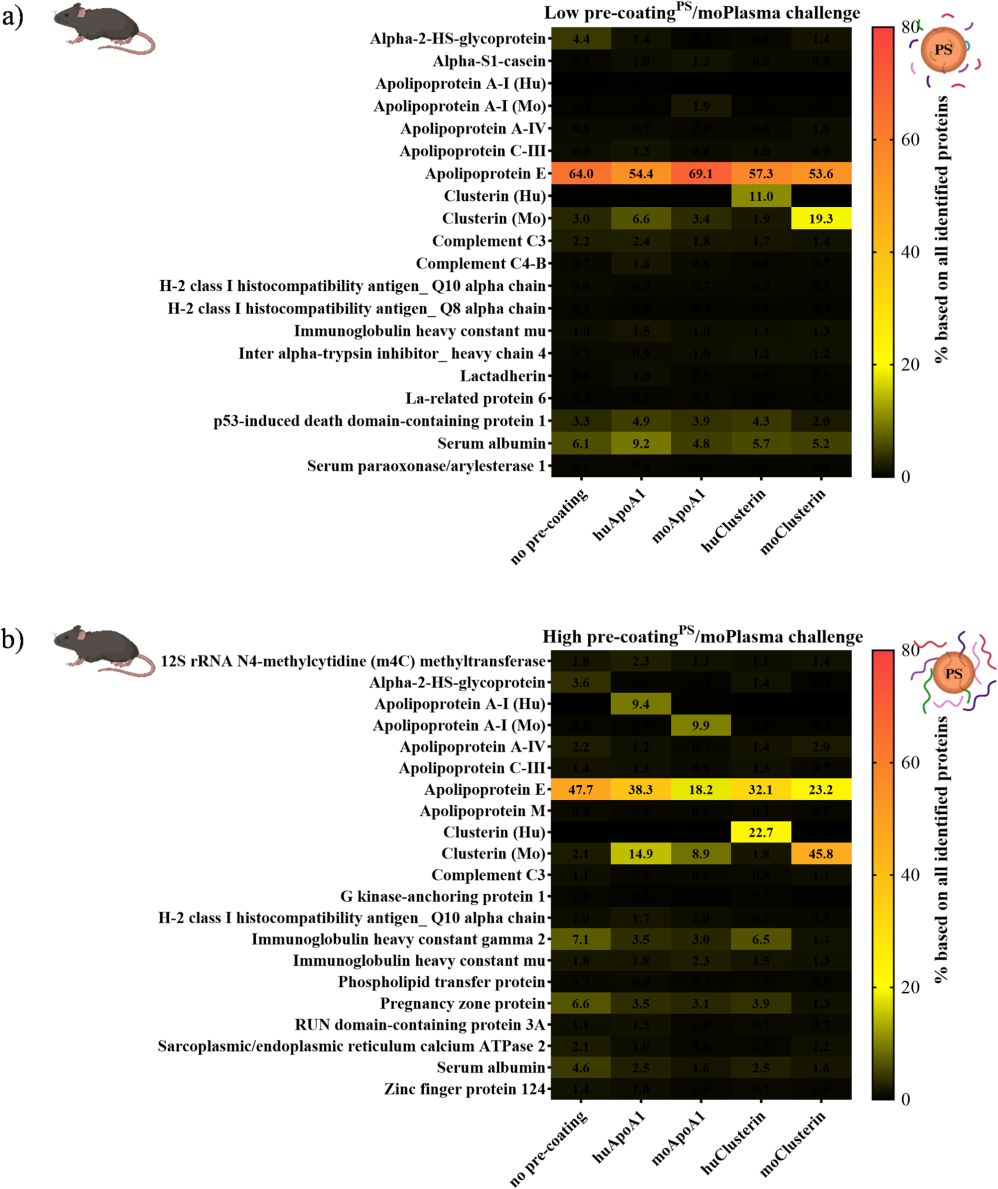
LC-MS analysis of corona composition: pristine and ApoA1 or clusterin **(a)** Low: 30 µg, **(b)** High: 120 µg, per 0.05 m2 surface area of pre-coated PS NPs, and then challenge with mouse plasma

For cellular uptake studies, we used a mouse and a human macrophage cell line, respectively. When we precoated unfunctionalized PS-NPs with different amounts of the single proteins ApoA1 and clusterin from human and mouse sources, we observed that the uptake of phagocytic particles by murine macrophages decreased with increasing amount, especially at high pre-adsorption levels of 120 µg ApoA1 (*p* < 0.0001) or clusterin (*p* < 0.0001) per 0.05 square meter of NP surface area (Fig. 3a). Consistently, we did not observe a stealth effect in murine macrophages under low precoating conditions after PBS challenge (Fig. 3b), but did observe a stealth effect with high precoating treatment (*p* < 0.0001) (Fig. 3c). Whereas after exposure to human plasma, further recruitment of similar plasma proteins through protein-protein interactions, in particular huApoA1 and huClusterin (Fig. 4a-b), led to a more pronounced reduction in cellular uptake (Fig. 3b-c). On the contrary, exposure to mouse plasma enhanced cellular uptake by mouse macrophages due to the leading position of another major protein component, apolipoprotein E, in their overall compositional patterns (Fig. 5a-b). Given that low-density lipoprotein (LDL) receptor-associated protein (also known as α2-macroglobulin receptor) selectively recognize apolipoprotein E in the liver, where macrophages mainly reside, lipoprotein and scavenger receptors are the crucial driving factors for strong interactions with macrophages [27, 28]. The uptake results observed for PS NPs were also applied to PS-NH2 NPs (Figure S4a-b). These results illustrate that for PEGylated particles, adsorption of a certain amount [39] of stealth protein is a necessary prerequisite to maintain the stealth functionality of nanocarriers in plasma.

In order to generate human macrophages as comparison for murine macrophages, we differentiated human monocytes which were isolated from peripheral blood mononuclear cells (PBMC) to produce human macrophages [40]. After incubation with macrophage colony-stimulating factor (M-CSF) for 6 days, cells were elongated and vacuolated [41] (Figure S6a). In addition, the expression of CD14 and CD16 was significantly upregulated in the M-CSF-treated group, but not in the non-M-CSF-treated group, further confirming successful differentiation into macrophages (Figure S6b). When the overall protein composition pattern is determined by ApoA1 or clusterin (Fig. 4a-b, S13a-b), murine and human macrophages show the same effect (Fig. 3b-e, S4a-b, S6c), but differ in their response to the predominant pattern of apolipoprotein E (Fig. 5a-b, S14a-b). More specifically, after mouse plasma challenge, we observed a decrease in cellular uptake by human macrophages, but not by murine macrophages (Fig. 3b-e, S4a-b, S6c), suggesting that apolipoprotein E plays an opsonic role in murine macrophages, whereas dysopsonin activity is present in human macrophages. In addition, the enrichment of vitronectin and apolipoprotein E on PS-COOH NPs caused by carboxyl functionalization (Figure S15a-b, S16a-b) further confirmed that the composition of the protein corona is largely determined by the surface properties of the particles themselves. And the corresponding cellular uptake of PS-COOH NPs by murine (Figure S5a-b) and human macrophages (Figure S6d) did not significantly decrease, but even increased in some cases. This may be due to interference from the opsonic activity of vitronectin [10, 42].

**Fig. 6:**
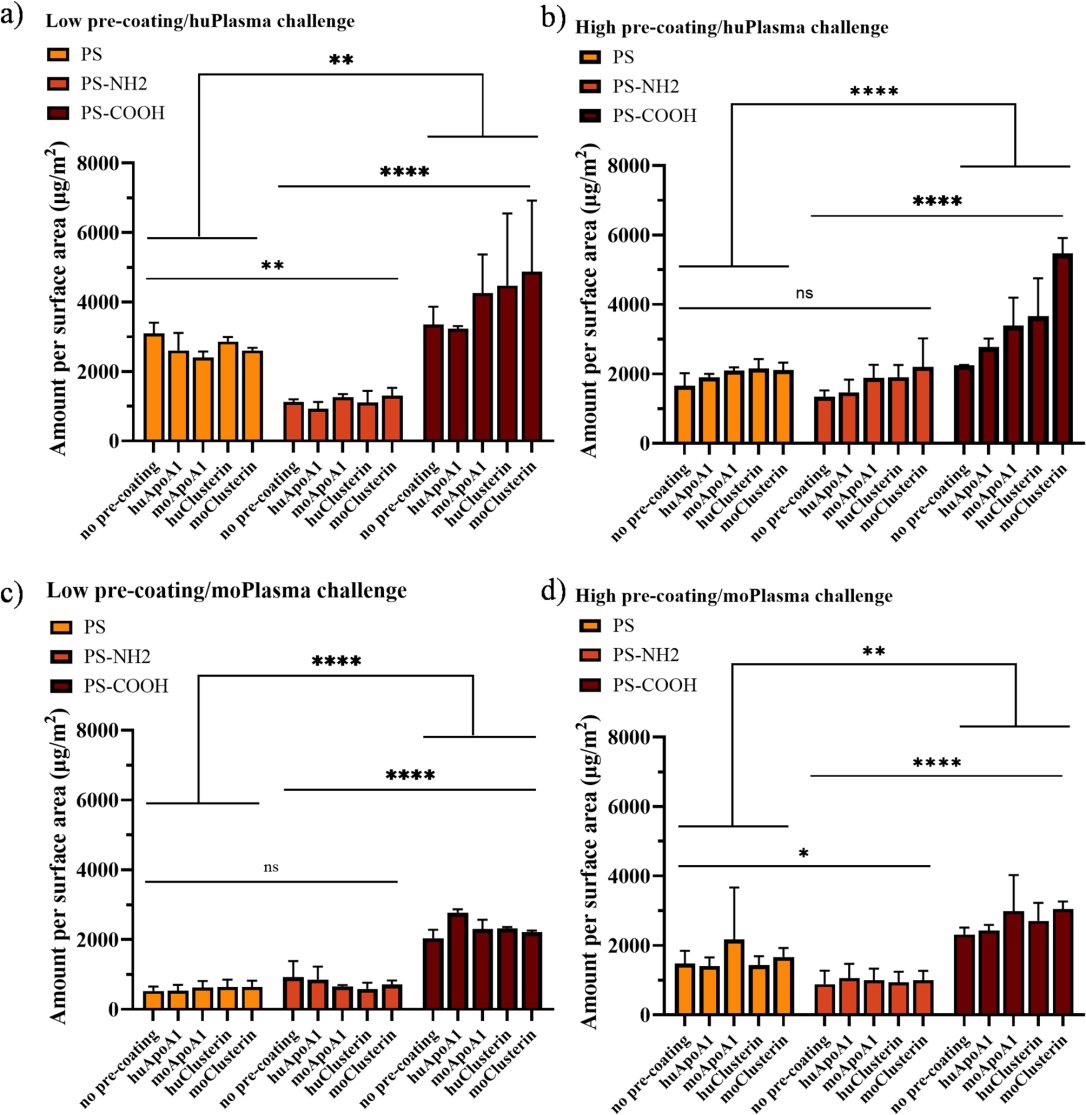
Quantitative characterization of hard protein corona patterns on three polystyrene nanoparticles (plain, amino-functionalized and carboxyl-functionalized) using a Pierce 660 nm protein assay. NPs (per 0.05 m2) were exposed to **a**,** c)** low: 30 µg, **b**,** d)** high: 120 µg of individual proteins (ApoA1 or clusterin) from human and mouse sources for 1 h at 37 °C. Precoated particles were challenged with a-b) huPlasma or **c-d)** moPlasma for 1 h at 37 °C. Proteins were blotted from the nanoparticle surface after multiple washings with SDS-Tris-HCl solution. All values are expressed as mean ± standard deviation (SD, *n* = 2)

As shown in the beginning, precoating altered the surface property of the particles and this could be observed in the final protein adsorption capacity (Fig. 6). In particular, the total protein affinity of human plasma (Fig. 6a-b) for the particles is significantly higher than that of mouse plasma (*p* < 0.0001) (Fig. 6c-d). And carboxyl-functionalized particles (PS-COOH) showed the highest adsorption amount due to their high negative charge, followed by plain particles (PS-NP), and relatively positively charged amino-functionalized particles (PS-NH2) adsorbed the least amount of protein (Fig. 6a, d). While the overall adsorption fluctuations between low and high dose protein pre-modification were noticeable among three different particles (Fig. 6a versus 6b; 6c versus 6d), this is due to the homogenization of the surface charge difference between functionalized and unfunctionalized particles by the protein pre-coating (Figure S1b, d, f). As a consequence, the electrostatic interactions between the pre-coated particles and the plasma proteins were also affected.

What’s more, this further recruitment by subsequent plasma exposure affects the outer layer of the protein corona, which is critical for interactions with biosystems and affects cellular uptake. To visualize and understand the complexity of the protein corona, we performed one-dimensional sodium dodecyl sulfate polyacrylamide gel electrophoresis (SDS-PAGE) and detected proteins by silver staining (Figure S7-S12). Typically, 2–6 dominant protein components were observed. For individual pre-coatings (Figure S7), 28 kD indicates ApoA1. Since clusterin is a 75–80 kD disulfide-linked heterodimeric protein, the disulfide link was cleaved after dithiothreitol (DTT) treatment, resulting in a 38 kD protein band corresponding to clusterin. In the context of human plasma (Figures S8a-c, S10a, S11a, and S12a), ApoA1 and clusterin are the most abundant components, together with a faint band for serum albumin at approximately 66 kD, whereas in the context of mouse plasma (Figures S9a-b, S10b, S11b, and S12b), two relatively thick bands at 36 kD indicate apolipoprotein E and 55 kD refers to vitronectin.

To identify proteins of lower abundance and to determine whether exchange and substitution have occurred, we applied label-free quantitative proteomics by liquid chromatography-mass spectrometry (LC-MS). We observed that selectively pre-coated proteins still secure different levels of enrichment after whole plasma challenge, depending on their initial individual pre-coating dose (Figs. 4a-b and 5a-b, S13a-b, S14a-b, S15a-b, and S16a-b). In particular, when a high dose of individual protein pre-adsorption is applied, ApoA1 and clusterin remarkably outnumber all other protein components in abundance (Figs. 4b and 5b, S13b, S14b, S15b, and S16b), indicating a strong affinity and stability of the initially formed protein corona. Furthermore, it shows that there is no apparent exchange or substitution of the pre-formed protein coating after exposure to total plasma. However, when these pre-coated particles were subsequently exposed to mouse plasma (Fig. 5a-b, S14a-b, and S16a-b), the most abundant protein detected was apolipoprotein E. The low amount of protein used in the pre-coating did not completely cover the surface, allowing more proteins (in this case mainly apolipoprotein E) to adhere (Fig. 5a, S14a, and S16a). In contrast, the high precoating treatment still retained a large amount of the selective protein (ApoA1 or clusterin) in the protein corona (Fig. 5b, S14b and S16b). This result emphasizes that the external protein source1 [20, 21] is also critical for the formation of the protein corona.

Although albumin is the most dominant component in both human and mouse plasma, accounting for approximately 60% of the total protein abundance (Figure S17a-b), it ultimately failed to compete with lipoproteins due to its low affinity for particles (Figure S18-S20). This phenomenon is also known as the Vroman effect [43–45]as we analyzed the protein corona after a relatively long incubation time (1 h), which is already sufficient to form a stable protein corona [46, 47]. In addition, pre-adsorption of varying amounts of ApoA1 or clusterin (both categorized as lipoproteins) didn’t change the overall classification pattern within the same particle type, but showed only slight changes between the human and mouse plasma challenge systems (Figure S18-S19: huPlasma Challenge versus moPlasma Challenge), confirming once again that the composition of the protein corona is primarily determined by the surface properties and the external protein source. In this case, lipoproteins are the largest category in the final complex protein mixture. Compared to plain and amino functionalized particles, carboxyl-modified particles adsorbed the least amount of lipoproteins, and their proportion in the mouse plasma challenge system is also lower than that in human plasma (Figure S19e *versus* S19f, S20e *versus* S20f). Accordingly, we expect a correlation between high lipoprotein content and low macrophage interactions [48, 49]which is consistent with our previous cellular uptake results. We want to point out that stealth is the basis for a long circulation time in vivo, but it is only helpful if further targeting e.g. by antibodies, nanobodies, or sugar motifs etc. are build upon this.

## Conclusion

In this study, we determined the efficacy of protein precoating of nanocarriers across interspecies protein sources and investigated potential differences in cellular uptake by human and murine macrophages. The composition of the protein corona is strongly determined by the surface properties of the particles themselves as well as the external protein sources [20]. We demonstrated that selective attachment of stealth proteins by pre-coating can be stable even after challenge with normal plasma, thereby maintaining the stealth functionality of nanocarriers and subsequently reducing cellular uptake by macrophages. We observed a large discrepancy between these interspecies experiments, largely due to the diverse protein corona compositions [50, 51]. These results suggest that the impact of interspecies differences in protein, plasma, and cell functions must be systematically evaluated to overcome barriers to translation from mouse and human sources.

## Materials and methods

### Materials

Dulbecco´s phosphate buffered saline (DPBS) without calcium and magnesium was purchased from Gibco/life technologies, Germany. Human Apolipoprotein AI (ApoAI) Native Protein was purchased from MyBioSource, USA. Recombinant Mouse Apolipoprotein A I was purchased from abcam, Netherland. Clusterin Human NATIVE (Apolipoprotein J, Apo J) was purchased from BioVendor, Germany. Recombinant Mouse Clusterin was purchased from BioLegend, Netherland. Histopaque 1077 (Sigma-Aldrich) was purchased from Merck, Germany. Recombinant human M-CSF was purchased from abcam, Netherland. PE anti-human CD14 and APC anti-human CD16 antibody were purchased from BioLegend, USA.

### Synthesis of polystyrene nanoparticles

Polystyrene nanoparticles (PS-NP) stabilized with the non-ionic surfactant Lutensol AT50 (BASF, Ludwigshafen) were synthesized as previously reported [52]. The fluorescent dye Bodipy [53] was in-cooperated for cellular uptake studies. Briefly, nanoparticles were synthesized via free-radical copolymerization miniemulsion technique [31]. For amino-functionalized nanoparticles 2-aminoethyl methacrylate hydrochloride (AEMH, 90%, Sigma-Aldrich) was used whereas for carboxy-functionalized nanoparticles acrylic acid AA (99%, Aldrich) was chosen.

### PS-NP

The macro emulsion was composed of an aqueous continuous phase containing Lutensol AT50 (25%wt. in water) in sterile water from Ampuwa (Fresenius Kabi, Germany). The organic dispersed phase contained distilled styrene, BODIPY 523/535 as fluorescent dye, and 2,2′-Azobis(2-methylpropionamidine) dihydrochloride in hexadecane. The continuous phase was added slowly to the dispersed phase under constant stirring. The macro emulsion was stirred for 1 h at high speed. The emulsion was then passed through a microfluidizer (Microfluidics USA, LM10). The miniemulsion obtained was directly transferred into a flask and let to polymerize under stirring condition for 18 h at 72 °C. The dispersion was purified by successive centrifugations at 20,000 g. Half of the supernatant was removed and replaced by sterile water from Ampuwa (Fresenius Kabi, Germany). The purification step was repeated four times.

### PS-NH2

The same procedure as for PS-NP was followed, 2-aminoethyl methacrylate hydrochloride (AEMH, Sigma Aldrich) was used as a co-monomer. It was dissolved in the aqueous continuous phase containing Lutensol AT50. The dispersion was purified by successive centrifugations at 20,000 g. Half of the supernatant was removed and replaced by sterile water from Ampuwa (Fresenius Kabi, Germany). The purification step was repeated four times.

### PS-COOH

The same procedure as for PS-NP was followed, acrylic acid was used as a co-monomer along with styrene in the disperse phase. The dispersion was purified by successive centrifugations at 20,000 g. Half of the supernatant was removed and replaced by sterile water from Ampuwa (Fresenius Kabi, Germany). The purification step was repeated four times.

### Circular dichroism spectroscopy (CD)

CD measurements were carried out in a JASCO 1500 Circular Dichroism (Jasco Inc., Easton, MD) with a 1 mm path length quartz cell (HELLMA, Germany) at room temperature. Native human and mouse plasma (0.1 mg/mL) served as controls, while protein-coated polystyrene (PS) nanoparticles (0.5 mg/mL) were suspended in PBS. Prior to CD measurements, nanoparticles were incubated with human or mouse plasma (1 h, 37 °C) and washed once to remove unbound proteins. All CD spectra were recorded at wavelengths ranging from 190 to 260 nm and each spectrum was an average of 3 scans. Data were fit using SELCON3, CONTINLL, and CDSSTR method run through CDPro to determine the secondary structure contents including helix, strand, turns, and unordered coil [54–56].

### Dynamic light scattering (DLS) and zeta ζ potential

Nanoparticles (40 ug/mL) suspended in Milli-Q water were transferred into a borosilicate glass cuvette and measured at 25 °C with a 90° scattering angle using a Zetasizer Nano Z (Malvern Panalytical). Zeta potential measurements were conducted using disposable folded capillary cells. For measurements of nanoparticles with a protein corona, the samples were prepared as described below, meaning that free plasma proteins were removed before the measurements.

### Multi-angle dynamic light scattering

Multi-angle dynamic light scattering experiments were performed with an ALV-CGS 8 F SLS/DLS 5022 F goniometer equipped with eight simultaneously working ALV 7004 correlators, eight QEAPD Avalanche photodiode detectors and a HeNe laser (632.8 nm, 25 mW output power) as light source at 37 °C. Nanoparticle dispersions (1 µl, 10 mg/mL) were measured in 1 mL of filtered Dulbecco`s magnesium- and calcium-free phosphate buffered saline (PBS) buffer solution (GIBCO, Invitrogen). Plasma was filtered through a Millex GS 220 nm filters (Millipore) into cylindrical quartz cuvettes (20 mm diameter, Hellma, Müllheim) and nanoparticles were directly (1 µL) to the cuvette and incubated with human serum for 1 h at 37 °C before the measurement. Scattering angle include 30°,50°,70°,90°,110°,130°, and 150°. Scattering angle of 30° was exhibited as representative to evaluate the aggregation behavior of particles. Data was analyzed according to the method from Rausch et al. [34].

### Cryo-TEM

10 µl of the sample was placed onto a 400 mesh copper grid covered with lacey film. The excess dispersion was removed by blotting with filter paper. The grid is plunged into liquid ethane (automated plunging system, Vitrobot FEI) and transferred in liquid nitrogen to the TEM. Prior to the preparation, the grids were treated with oxygen plasma to make the film hydrophilic.

### Transmission electron microscopy (TEM)

Two microliters of each sample were placed on a lacey grid. Then, the samples were embedded in 1% trehalose containing 4% uranyl acetate to increase contrast.

### Evaluation of pre-coating efficacy in plasma challenge

Polystyrene nanoparticles (plain, amino-functionalized, and carboxyl-functionalized) were respectively pre-incubated with individual single ApoA1 or clusterin (Low: 30 µg, High: 120 µg, per 0.05 m [2] surface area) at 37℃ for 1 h to form pre-coating. Unbound proteins were removed and washed one time via centrifugation. Then challenge pre-coated particles with human plasma or mouse plasma for 1 h, 37℃ to allow corona formation. Wash one time again to remove unbound proteins for further use in cell uptake experiments.

### Blood plasma

Human plasma was obtained from the Department of Transfusion Medicine Mainz from healthy donors in accordance with the Declaration of Helsinki and stored at -80 °C. Innovative Grade™ US Origin C57BL6 Mouse Plasma was purchased from Innovative Research, which is recovered from whole blood donations from normal, healthy mice of US Origin only. The blood is collected and processed into plasma, which remains frozen and is shipped on dry ice for maximum stability. In both cases, citrate was added as anti-coagulant. Before usage, plasma was centrifuged at 20 000 g (4 °C, 30 min) to remove protein aggregates.

### Protein corona preparation

As previously described a constant ratio between nanoparticle surface area and plasma was chosen [6] (0.05 m [2] PS-NP per 1 mL plasma). The dispersion was incubated at 37 °C, 300 rpm for 1 h and subsequently centrifuged (20 000 g, 1 h, 4 °C). The nanoparticle pellet was washed with PBS (3 times, 1 mL) to remove loosely and unbound proteins. For protein identification, the nanoparticle pellet was resuspended in 100 µL 2% SDS with 62.5 mM Tris hydrochloride solution and incubated for 5 min at 95 °C. The dispersion was centrifuged and the resulting supernatant contained desorbed corona proteins.

### Pierce 660 nm protein quantification assay

The protein concentration was determined by Pierce 660 nm protein Assay according to the manufactures´ instruction. Bovine serum albumin was used as standard. Absorbance was measured with a Tecan infinite plate reader.

### SDS polyacrylamide gel electrophoresis (SDS-PAGE)

Proteins were analyzed by SDS-PAGE using NuPage 10% Bis-Tris Protein Gels. 2 µg of protein was applied if gels were stained with Pierce Silver Staining Kit. The protein sample volume was adjusted with water to a volume of 26 µL and mixed with 4 µL of NuPage Sample Reducing Agend and 10 µL of NuPage LDS Sample Buffer. Electrophoresis was carried out for 1 h at 100 V. Gels were stained with Pierce Silver Staining Kit according to the manufactures´ instruction.

### In solution digestion

Proteins were precipitated using ProteoExtract protein precipitation kit according to manufactors´ instruction and in solution digestion was performed as pervious reported [57]. For LC-MS measurements, samples were diluted with 0.1% formic acid and spiked with 50 fmol/µl Hi3 EColi Standard (Waters Corporation) for absolute peptide quantification.

### Liquid chromatography-mass spectrometry (LC-MS)

A Synapt G2-Si mass spectrometer coupled with a nanoACQUITY UPLC system was used for proteomic experiments. The system was operated as described in several reports. Data was processed and peptides were identified with Progenesis QI for Proteomics. Generated peptide masses were searched against a reviewed human protein sequence database downloaded from Uniprot. The database was modified with the sequence information of Hi3 Ecoli standard (Chaperone protein ClpB) for absolute quantification. For peptide identification, at least three assigned fragments are required. For protein identification, at least two assigned peptides and five assigned fragments are needed. Peptides with a score parameter less than 4 were rejected. Based on the TOP3/Hi333 approach, the amount of protein in fmol was generated.

### Generation of monocyte derived human macrophages

Human macrophages were generated from healthy human donors from buffy coat according to the local ethics committee and Declaration of Helsinki. The study was approved by the local ethics committee of the Landesärztekammer Rheinland-Pfalz (ref. no: 837.439.12 (8540-F)). Peripheral blood mononuclear cells (PBMCs) were isolated by standard Ficol separation. Monocyte were differentiated to macrophages using 50 ng/mL M-CSF for 6 days as described [58, 59].

#### Cell culture

The RAW264.7 cell lines were cultured in Dulbecco´s modified eagle medium (DMEM) supplemented with 10% FCS, 100 U/mL penicillin, 100 mg/mL streptomycin and 2 mM glutamine. Monocyte derived human macrophages were cultured in Roswell Park Memorial Institute (RPMI) 1640 medium supplemented with 20% FCS, 100 U/mL penicillin, 100 mg/mL streptomycin. Cells were grown in a humidified incubator at 37 °C and 5% CO2. The cell viability and count were determined with trypan blue by an automated cell counter (TC10, Bio-Rad).

### Cell uptake experiments by flow cytometry

RAW264.7 or differentiated human macrophages were seeded out in 24-well plates (150 000 cells per well) and kept overnight at 37 °C. Prior to cell uptake studies, the cell culture medium was exchanged to cell culture medium without FBS. NPs were pre-coated with ApoA1 or Clusterin sourced from human or mouse, centrifuged and washed with PBS, and then incubated with human or murine plasma for 1 h to allow protein corona formation, followed with PBS wash to remove the excess proteins. BODIPY signal of polystyrene nanoparticles (Ex523/Em536) was then measured using a Tecan Infinite plate reader to assess nanoparticle retention after washed once with PBS and ensure uniform nanoparticle amounts for each condition. Protein coated NPs were added to cell culture medium without FBS at a final concentration of 40 µg/mL and incubated with cells for 2 h at 37 °C. Cells were washed with PBS to remove unbound nanoparticles, detached using Trypsin-EDTA, centrifuged at 500 × g for 5 min, and resuspended in PBS. The BODIPY signal of the nanoparticles was recorded using the BL1 channel of the Attune™ Nxt (Thermo Fisher Scientific, Germany) Flow Cytometer with an excitation laser of 488 nm and a band-pass filter of 530/30 nm. Cell debris was excluded by selecting a cell population in an FSC/SSC scatter plot processing the data with Attune™ NxT Software. The uptake experiments were carried out twice as independent experiments and within an experiment, for each condition, the cells were seeded as a triplicate.

### Statistical analysis

GraphPad Prism was used for the statistical analysis. All data are expressed as mean ± SD. Each experiment was repeated at least three times, unless otherwise indicated. For comparing two experimental groups a two-sided student´s t-test was performed. Calculated p values were considered to be significant *p < 0.05, **p < 0.01, ***p < 0.001, ****p < 0.0001.

## Electronic supplementary material

Below is the link to the electronic supplementary material.

Supplementary Material 1

## Data Availability

Data is provided within the manuscript or supplementary information files. Further data can be obtained by reasonable request from the corresponding author.
