# Supplementary material for: Engineered protein corona sustains stealth functionality of nanocarriers in plasma
Source: J Nanobiotechnology. 2025 Jul 14;23:512. doi: 10.1186/s12951-025-03565-x (PMC12261846; doi:10.1186/s12951-025-03565-x)
Supplement: Supplementary file 1 — Supplementary Material 1 [file 12951_2025_3565_MOESM1_ESM.pdf]

Supplementary Material

Engineered Protein Corona Sustains Stealth Functionality of Nanocarriers in Plasma

Xueqing Zhang[1,2], Shutian Si[2], Ingo Lieberwirth[2], Katharina Landfester[2], Volker Mailänder[1,2]

[1] Department of Dermatology, University Medical Center Mainz, Langenbeckstraße 1, 55131 Mainz, Germany
[2] Max Planck Institute for Polymer Research, Ackermannweg 10, 55128 Mainz, Germany

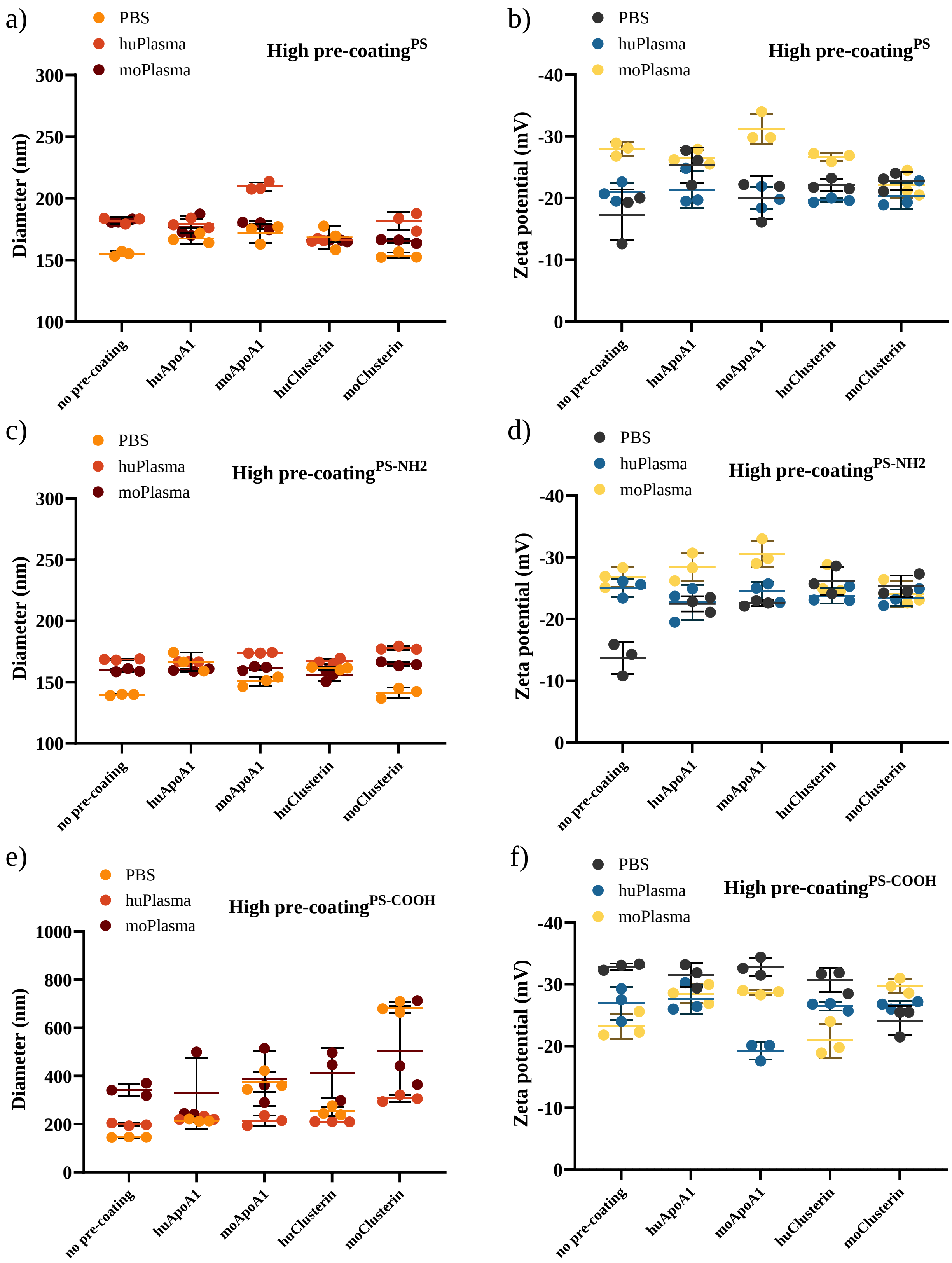

a)
PBS
huPlasma
moPlasma
High pre-coating^PS
Diameter (nm)
300
250
200
150
100
no pre-coating
huApoA1
moApoA1
huClusterin
moClusterin
b)
PBS
huPlasma
moPlasma
High pre-coating^PS
Zeta potential (mV)
-40
-30
-20
-10
0
c)
High pre-coating^PS-NH2
d)
High pre-coating^PS-NH2
e)
High pre-coating^PS-COOH
1000
800
600
400
200
0
f)
High pre-coating^PS-COOH

	PS					PS-NH2					PS-COOH				
	a	b	c	d	e	a	b	c	d	e	a	b	c	d	e
PBS	0.192	0.257	0.305	0.230	0.204	0.120	0.250	0.163	0.175	0.128	0.177	0.467	0.564	0.403	0.419
huPlasma	0.201	0.198	0.239	0.171	0.213	0.121	0.116	0.148	0.141	0.153	0.254	0.369	0.376	0.325	0.484
moPlasma	0.233	0.283	0.236	0.192	0.210	0.149	0.14	0.139	0.144	0.173	0.728	0.660	0.706	0.853	0.808

Polydispersity index (PDI): NPs were pre-coated with a) no pre-coating, b) huApoA1, c) moApoA1, d) huClusterin, e) moClusterin, and then challenged with human plasma or mouse plasma

Figure S1. Physico-chemical properties of a-b) plain, c-d) amino- and e-f) carboxyl-functionalized polystyrene nanoparticles. NPs were pre-coated with ApoA1 or clusterin, and then challenged with human plasma or mouse plasma. The size refers to the newly formed NP-protein complex after centrifugation and removal of the supernatant, but after 1st wash. Obtained hydrodynamic diameter for the size from DLS together with the obtained polydispersity index (PDI) in the table from a cumulant fit at a scattering angle of 90°. Values are expressed as mean ± standard deviation (SD, n=3).

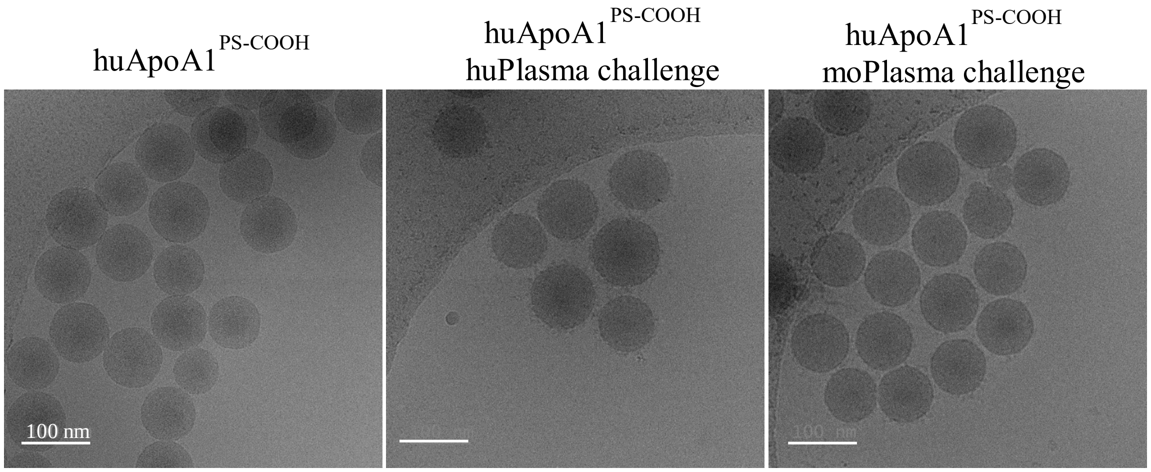

Figure S2. Cryo-TEM images of PS-COOH nanoparticles pre-coated with human ApoA1, incubated in different plasmas to reveal the structure of the protein corona. Samples were centrifuged and washed one time to remove unbound proteins. Scale bar: 100 nm.

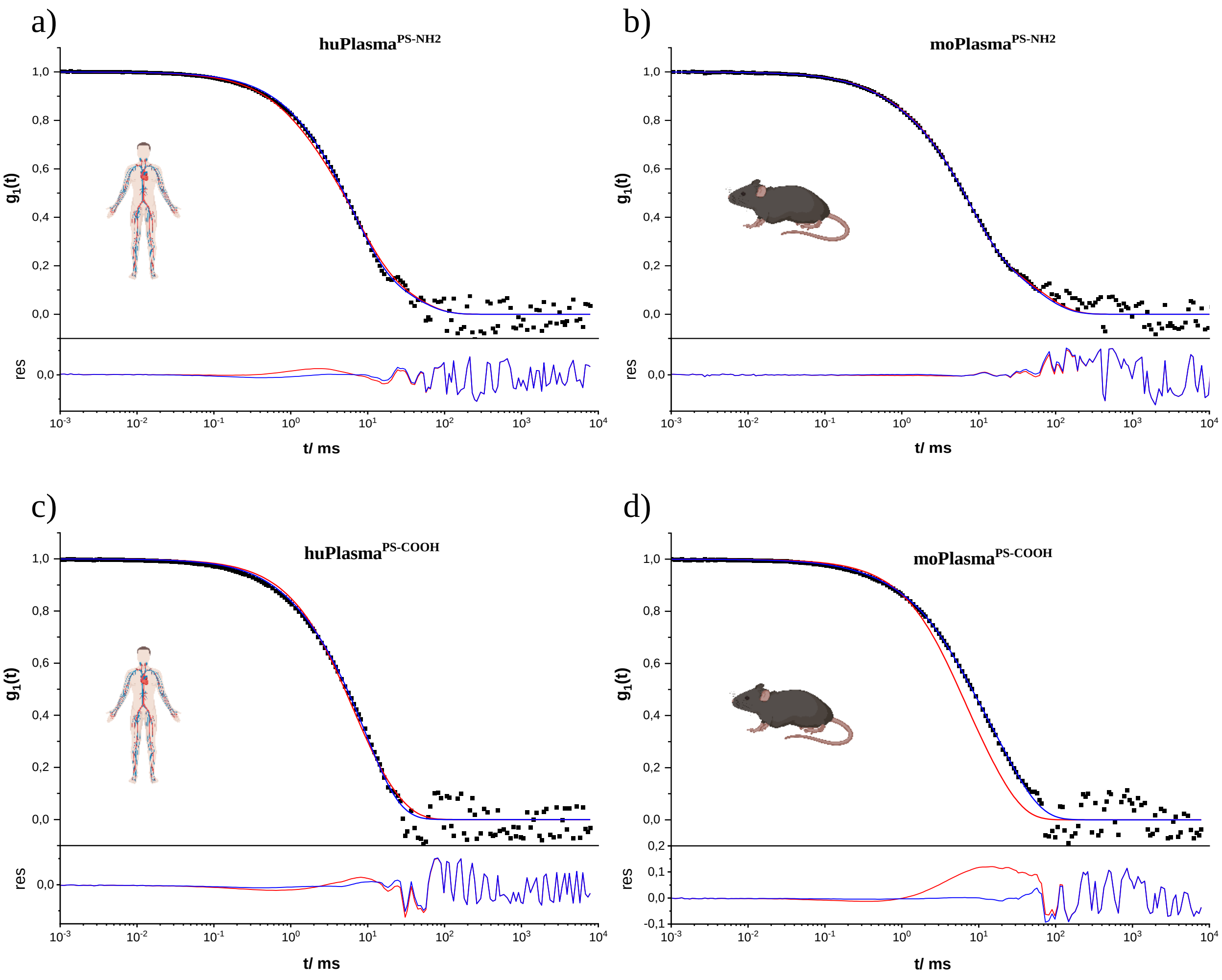

Figure S3. Determination of aggregation tendency of a-b) PS-NH2 and c-d) PS-COOH nanoparticles in concentrated human (left) or murine (right) plasma sources by dynamic light scattering (DLS). (Top) Exemplary autocorrelation functions (ACFs) at a scattering angle of 30° of nanoparticles in each plasma source together with the corresponding fits with (blue line) and without (red line) an additional aggregate term. (Bottom) Residuals resulting from the difference between the data points and both fits.

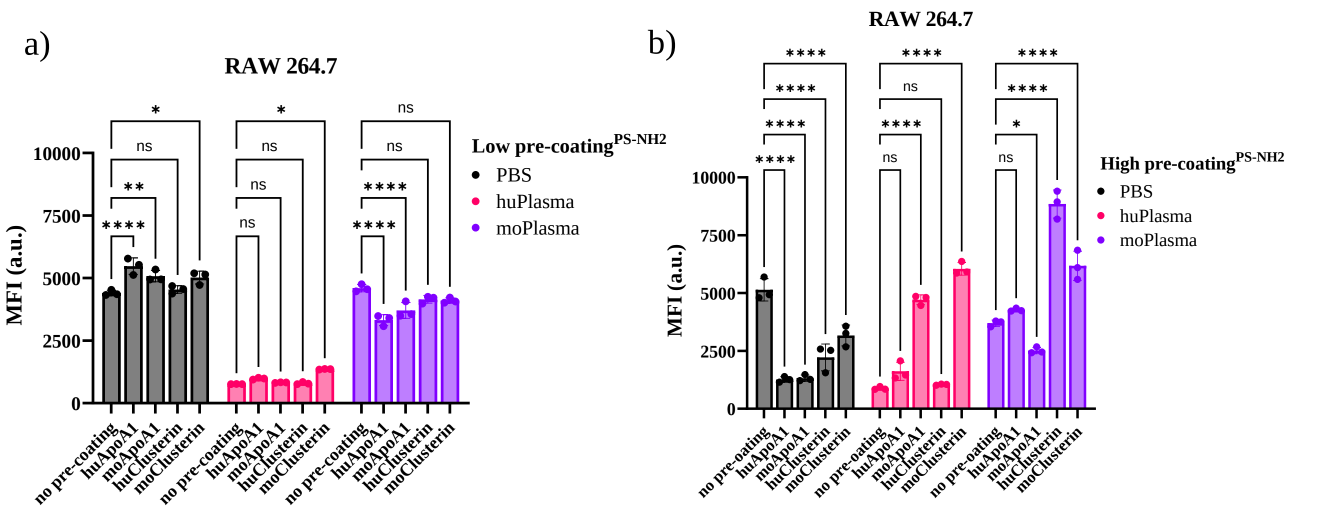

Figure S4. Flow cytometry analysis: RAW 264.7 cells were incubated with 40 μg/mL of particles for 2 h. Prior to cellular uptake studies, pristine and ApoA1 or clusterin a) Low: 30 μg, b) High: 120 μg, per 0.05 m^2 surface area pre-coated PS-NH2 NPs for 1 h, 37°C. Wash one time to remove unbound proteins. Then challenge pre-coated particles with human plasma or mouse plasma for 1 h, 37°C. Wash one time again to remove unbound proteins. Values are expressed as mean ± standard deviation (SD, n=3).

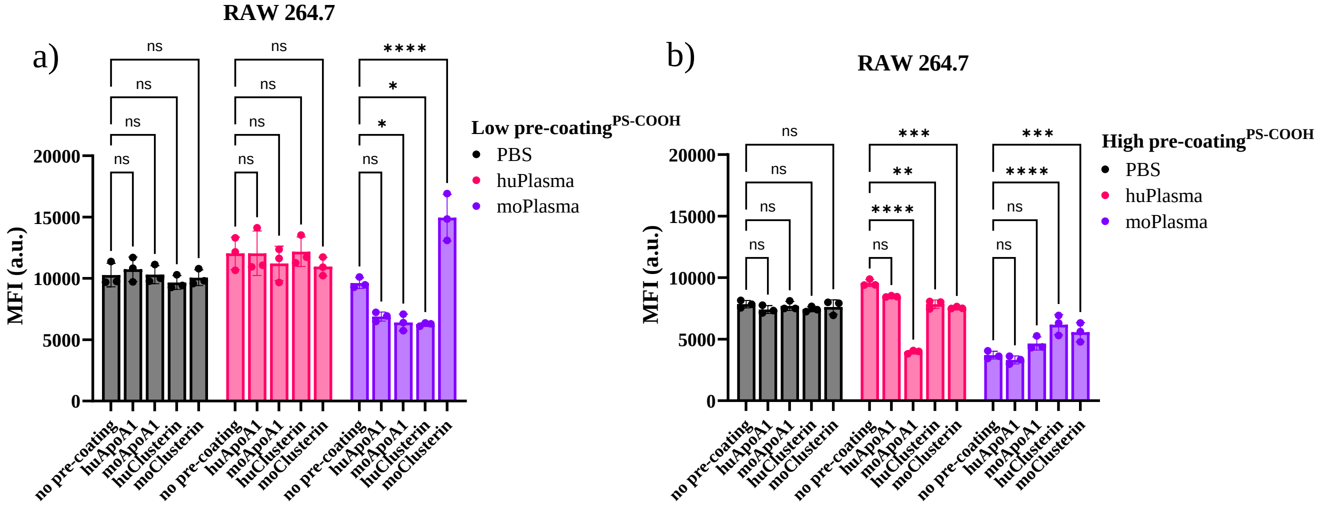

Figure S5. Flow cytometry analysis: RAW264.7 cells were incubated with 40 μg/mL of particles for 2 h. Prior to cellular uptake studies, pristine and ApoA1 or clusterin a) Low: 30 μg, b) High: 120 μg, per 0.05 m^2 surface area pre-coated PS-COOH NPs for 1 h, 37°C. Wash one time to remove unbound proteins. Then challenge pre-coated particles with human plasma or mouse plasma for 1 h, 37°C. Wash one time again to remove unbound proteins. Values are expressed as mean ± standard deviation (SD, n=3).

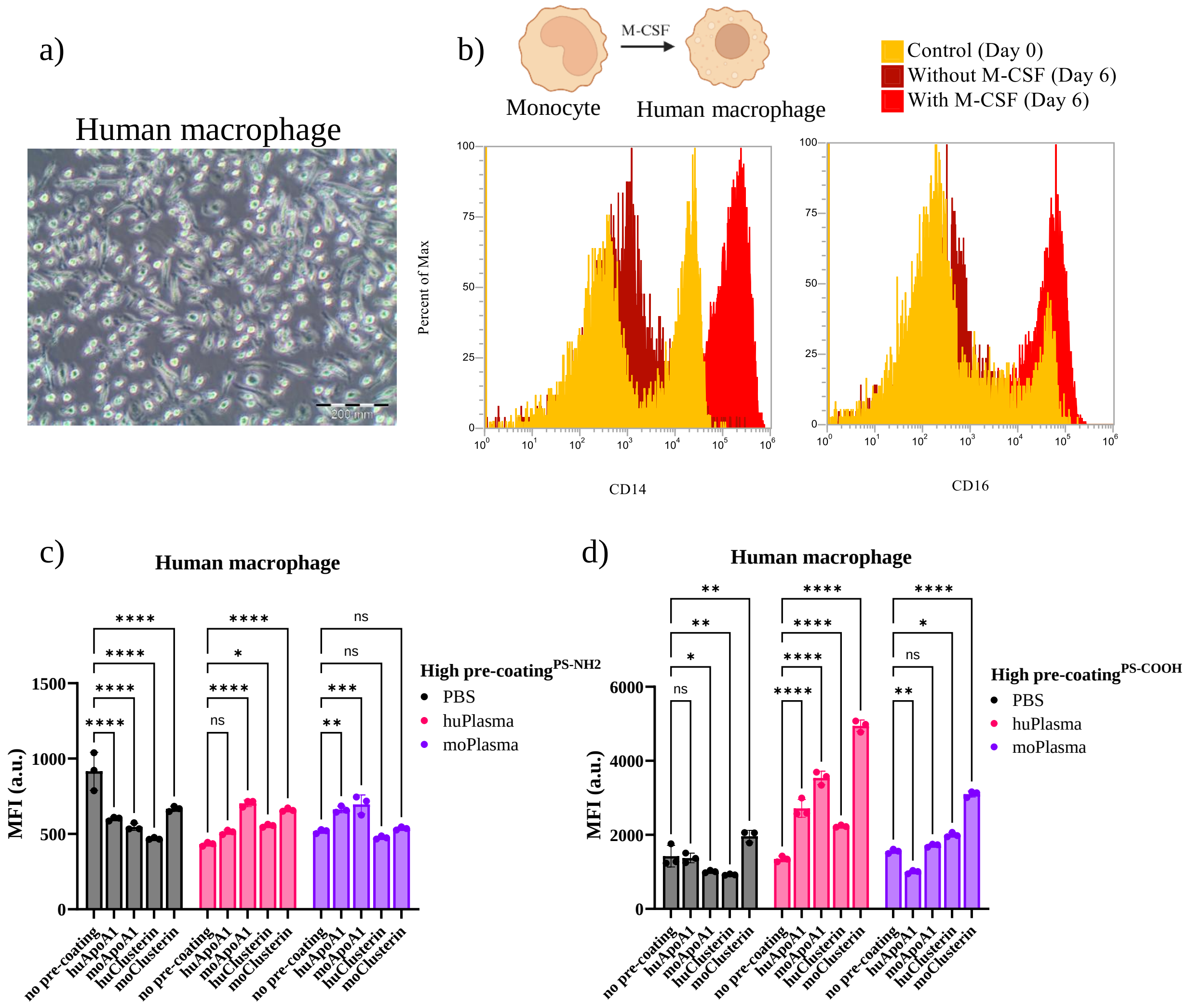

Figure S6. a) Morphology of differentiated macrophages. Scale bar = 200 mm b) Differential expression of macrophage marker CD14 and CD16. Human monocytes were differentiated for 6 days in the presence of M-CSF. Flow cytometry analysis: Human macrophages were incubated with 40 µg/mL of particles for 3 h. Prior to cellular uptake studies, pristine and ApoA1 or clusterin (High: 120 µg per 0.05 m^2 surface area) pre-coated c) PSNH2 or d) PS-COOH NPs for 1 h, 37°C. Wash one time to remove unbound proteins. Then challenge pre-coated particles with human plasma or mouse plasma for 1 h, 37°C. Wash one time again to remove unbound proteins. Values are expressed as mean ± standard deviation (SD, n=3)

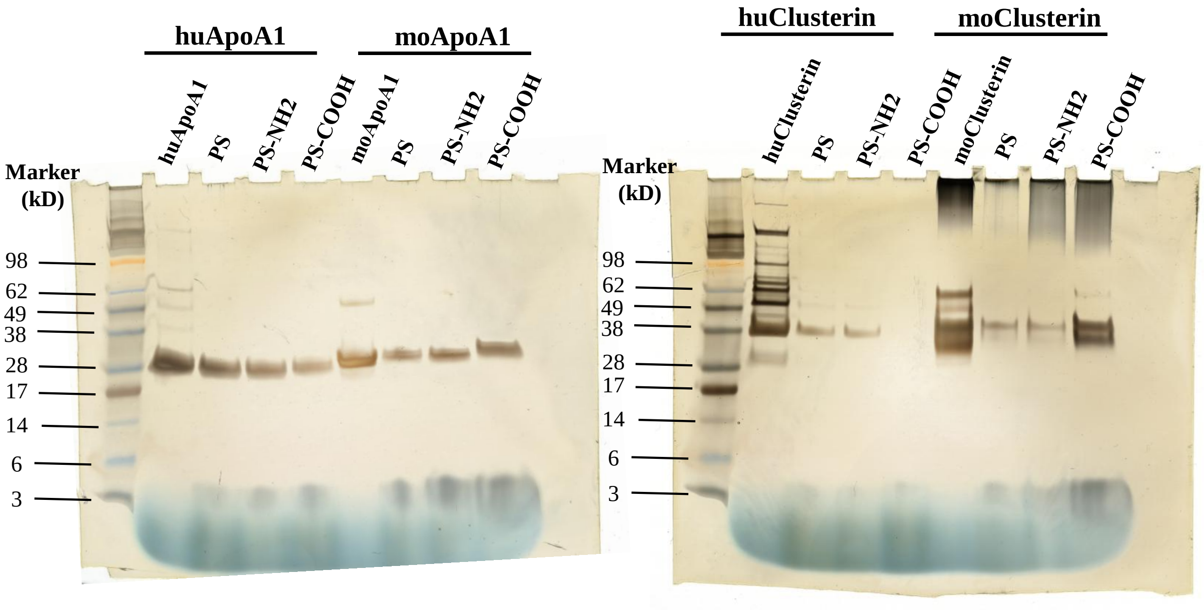

Figure S7. SDS PAGE analysis of the hard corona pattern of three different polystyrene nanoparticles (plain, amino- and carboxyl-functionalized). Pure ApoA1 and clusterin from both human and murine source serve as a reference. NPs were ApoA1 or clusterin (30 µg per 0.05 m^2 surface area) for 1 h, 37°C. Samples were isolated via centrifugation and washed three times to remove unbound proteins. Hard corona proteins were desorbed from the particles using 2% SDS, separated by SDS-PAGE and stained with a Silver Staining Kit.

a)

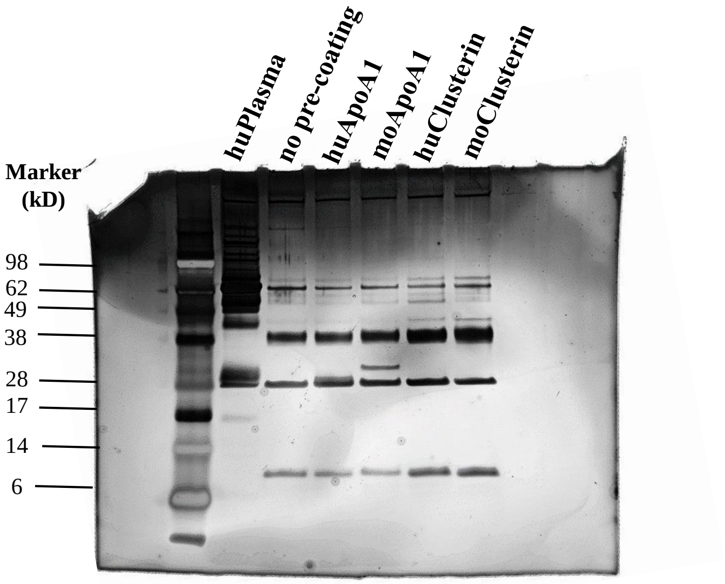

Low pre-coating^{PS}/huPlasma challenge

b)

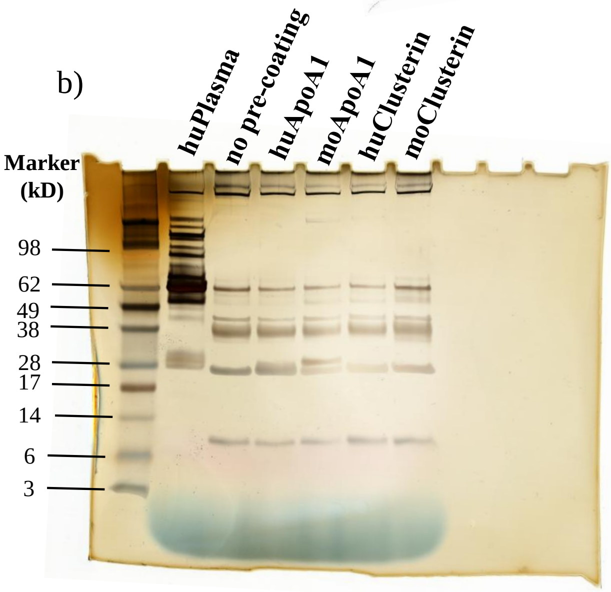

Low pre-coating$^{PS\text{-}NH2}$/huPlasma challenge

c)

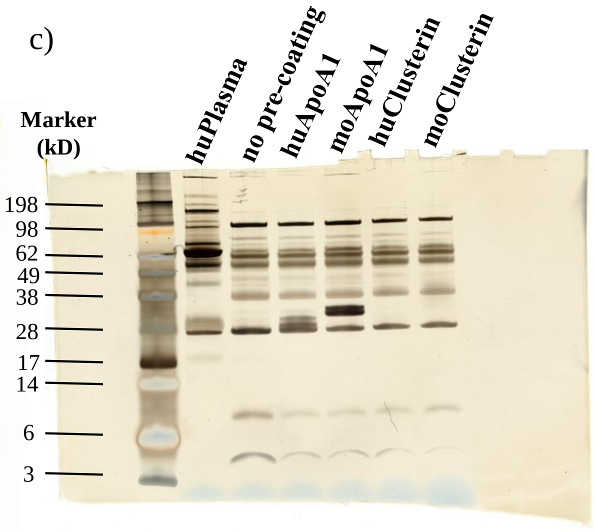

Low pre-coating$^{PS\text{-}COOH}$/huPlasma challenge

Figure S8. SDS PAGE analysis of the hard corona pattern of a) PS, b) PS-NH2 and c) PS-COOH nanoparticles. Human plasma serve as a reference. NPs were incubated with human plasma or pre-coated with ApoA1 or clusterin (30 µg per 0.05 m^2 surface area) from human or murine source, and then challenged with plasma. Samples were isolated via centrifugation and washed three times to remove unbound proteins. Hard corona proteins were desorbed from the particles using 2% SDS, separated by SDS-PAGE and stained with a Silver Staining Kit.

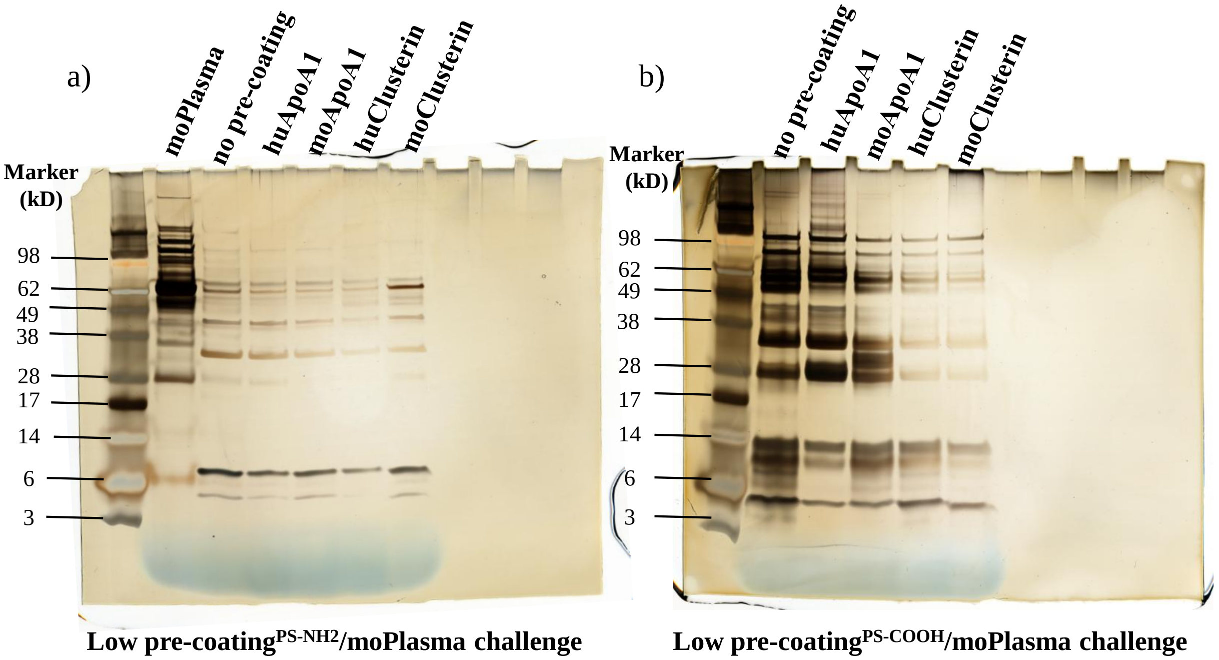

Figure S9. SDS PAGE analysis of the hard corona pattern of a) PS-NH2 and b) PS-COOH nanoparticles. Mouse plasma serve as a reference. NPs were incubated with mouse plasma or pre-coated with ApoA1 or clusterin (30 µg per 0.05 m^2 surface area) from human or murine source, and then challenged with plasma. Samples were isolated via centrifugation and washed three times to remove unbound proteins. Hard corona proteins were desorbed from the particles using 2% SDS, separated by SDS-PAGE and stained with a Silver Staining Kit.

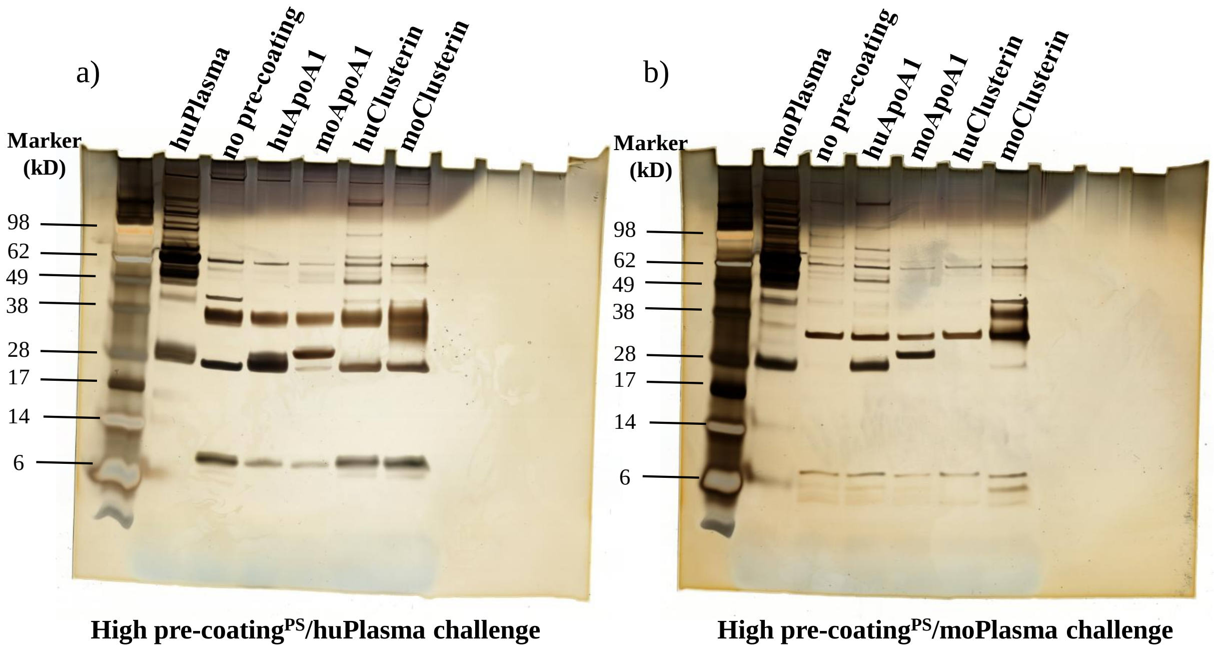

Figure S10. SDS PAGE analysis of the hard corona pattern of PS nanoparticles. Human plasma and mouse plasma serve as a reference. PS nanoparticles were incubated with human plasma, mouse plasma or pre-coated with ApoA1 or clusterin (120 µg per 0.05 m^2 surface area) from human or murine source, and then challenged with plasma. Samples were isolated via centrifugation and washed three times to remove unbound proteins. Hard corona proteins were desorbed from the particles using 2% SDS, separated by SDS-PAGE and stained with a Silver Staining Kit.

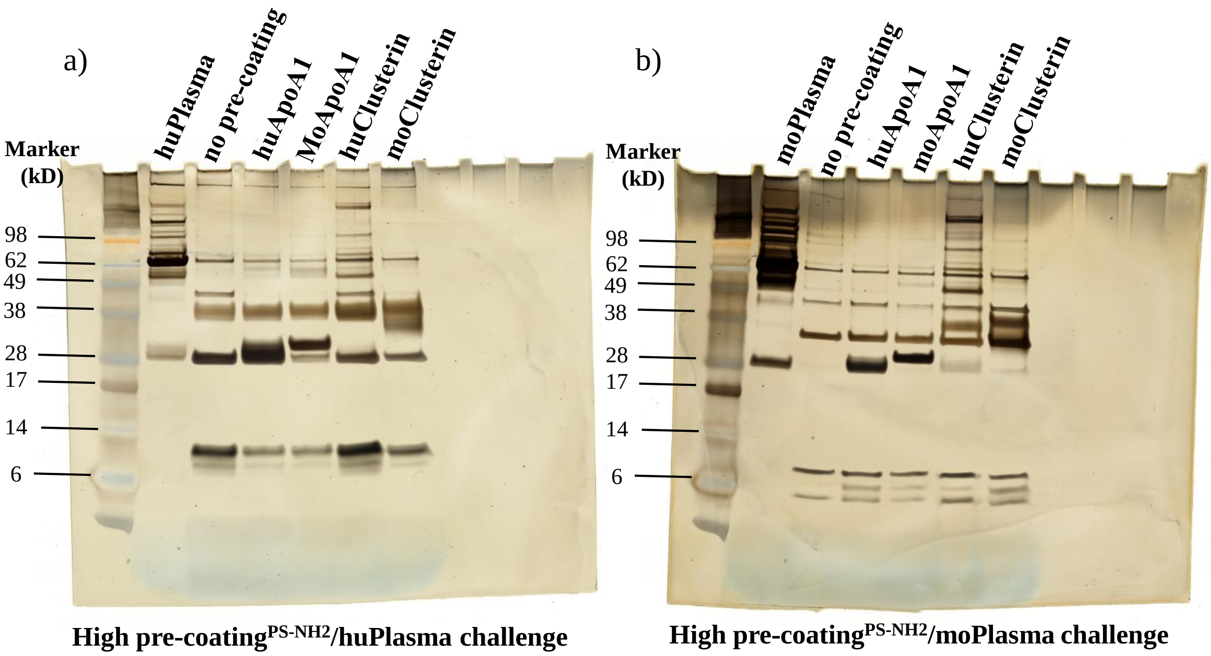

Figure S11. SDS PAGE analysis of the hard corona pattern of PS-NH2 nanoparticles. Human plasma and mouse plasma serve as a reference. PS nanoparticles were incubated with human plasma, mouse plasma or pre-coated with ApoA1 or clusterin (120 µg per 0.05 m^2 surface area) from human or murine source, and then challenged with plasma. Samples were isolated via centrifugation and washed three times to remove unbound proteins. Hard corona proteins were desorbed from the particles using 2% SDS, separated by SDS-PAGE and stained with a Silver Staining Kit.

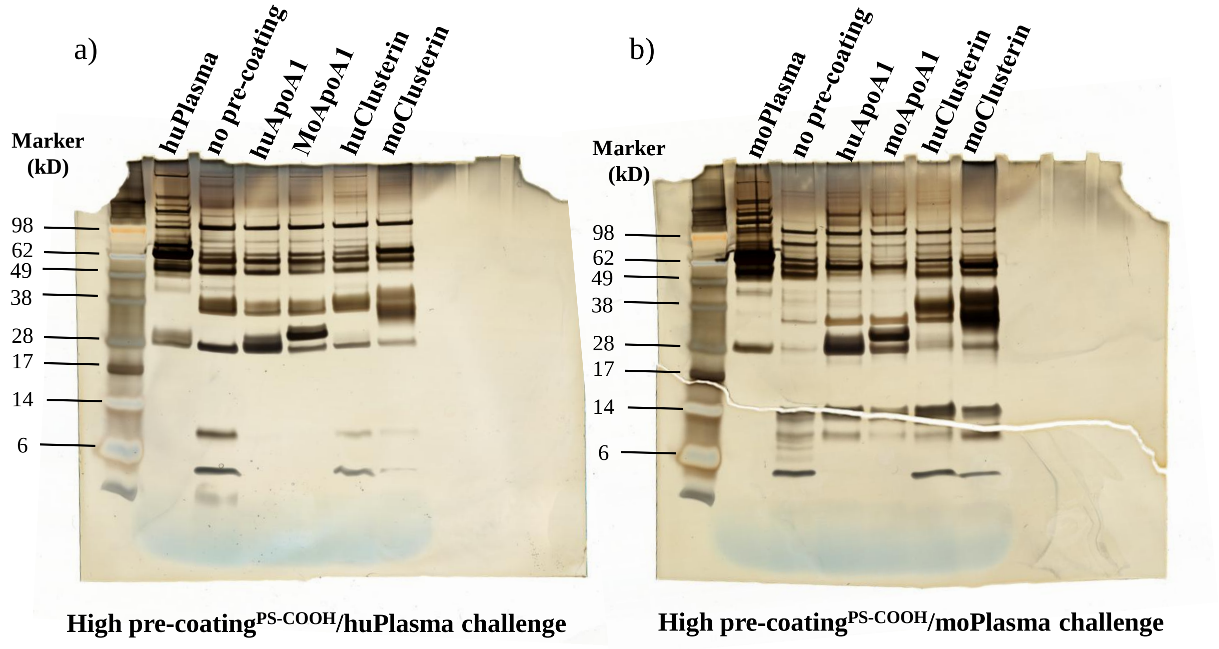

Figure S12. SDS PAGE analysis of the hard corona pattern of PS-COOH nanoparticles. Human plasma and mouse plasma serve as a reference. PS nanoparticles were incubated with human plasma, mouse plasma or pre-coated with ApoA1 or clusterin (120 µg per 0.05 m^2 surface area) from human or murine source, and then challenged with plasma. Samples were isolated via centrifugation and washed three times to remove unbound proteins. Hard corona proteins were desorbed from the particles using 2% SDS, separated by SDS-PAGE and stained with a Silver Staining Kit.

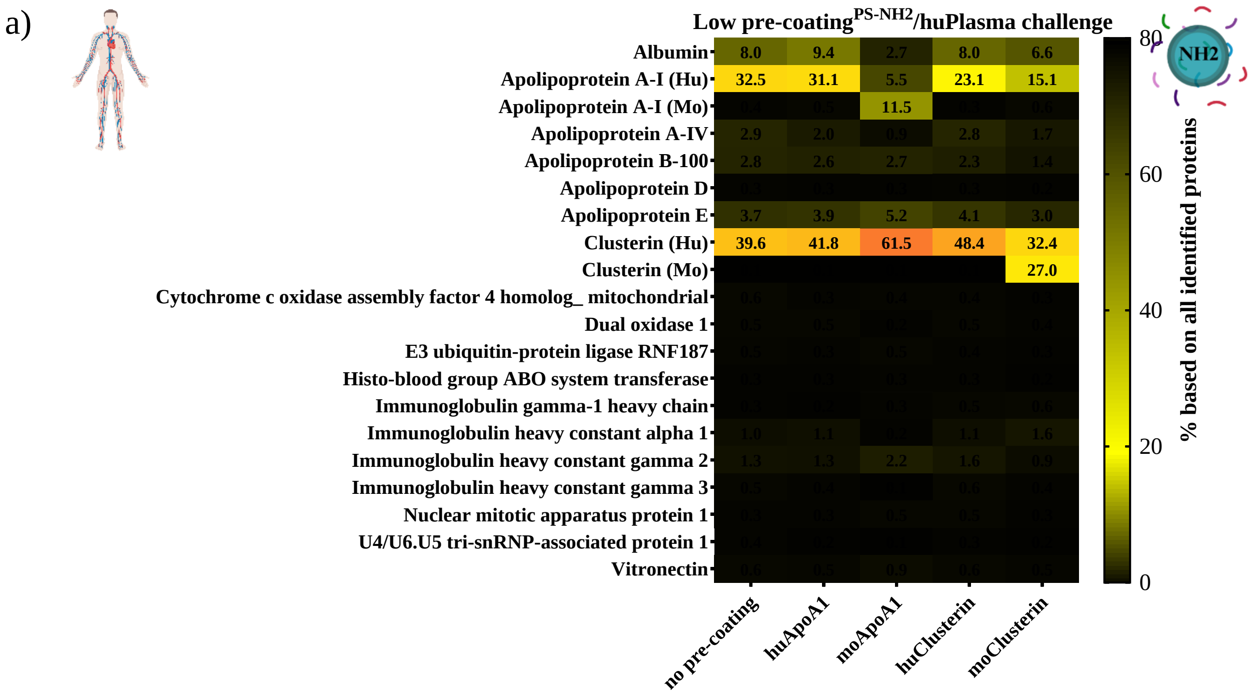

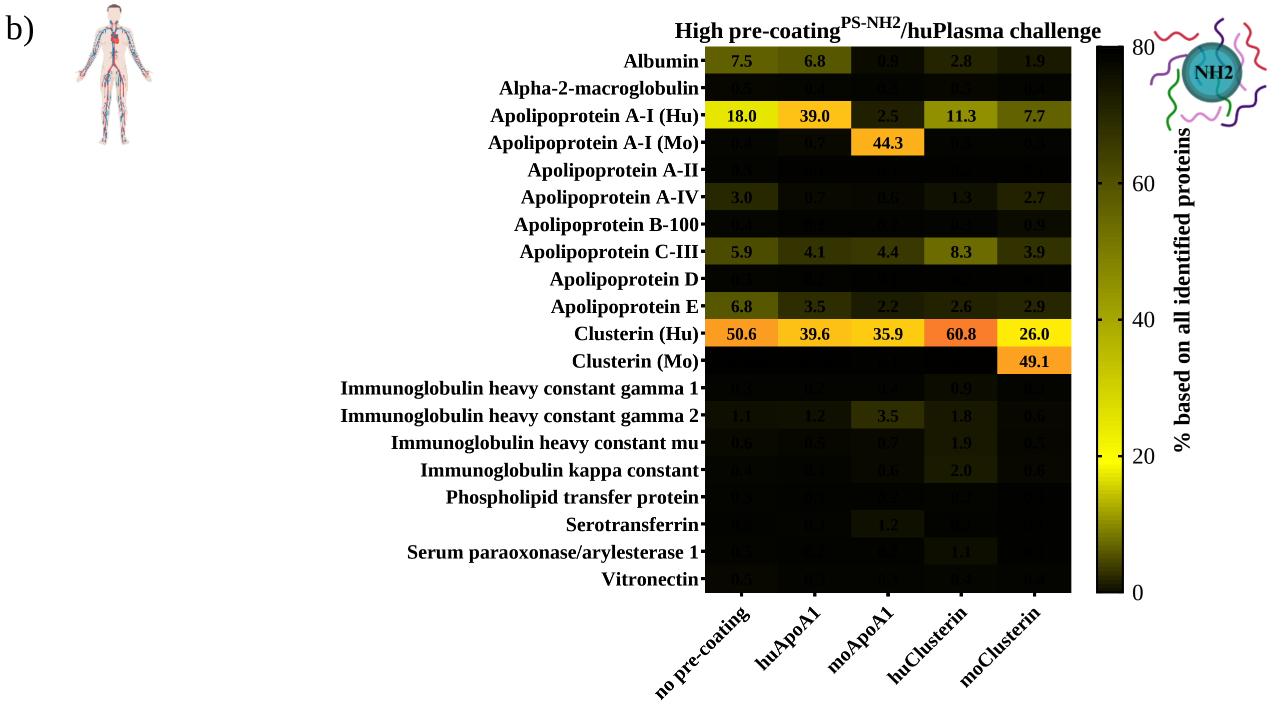

Figure S13. LC-MS analysis of the corona composition: pristine and ApoA1 or clusterin a) Low: 30 µg, b) High: 120 µg, per 0.05 m^2 surface area pre-coated PS-NH2 NPs, and then challenge with human plasma.

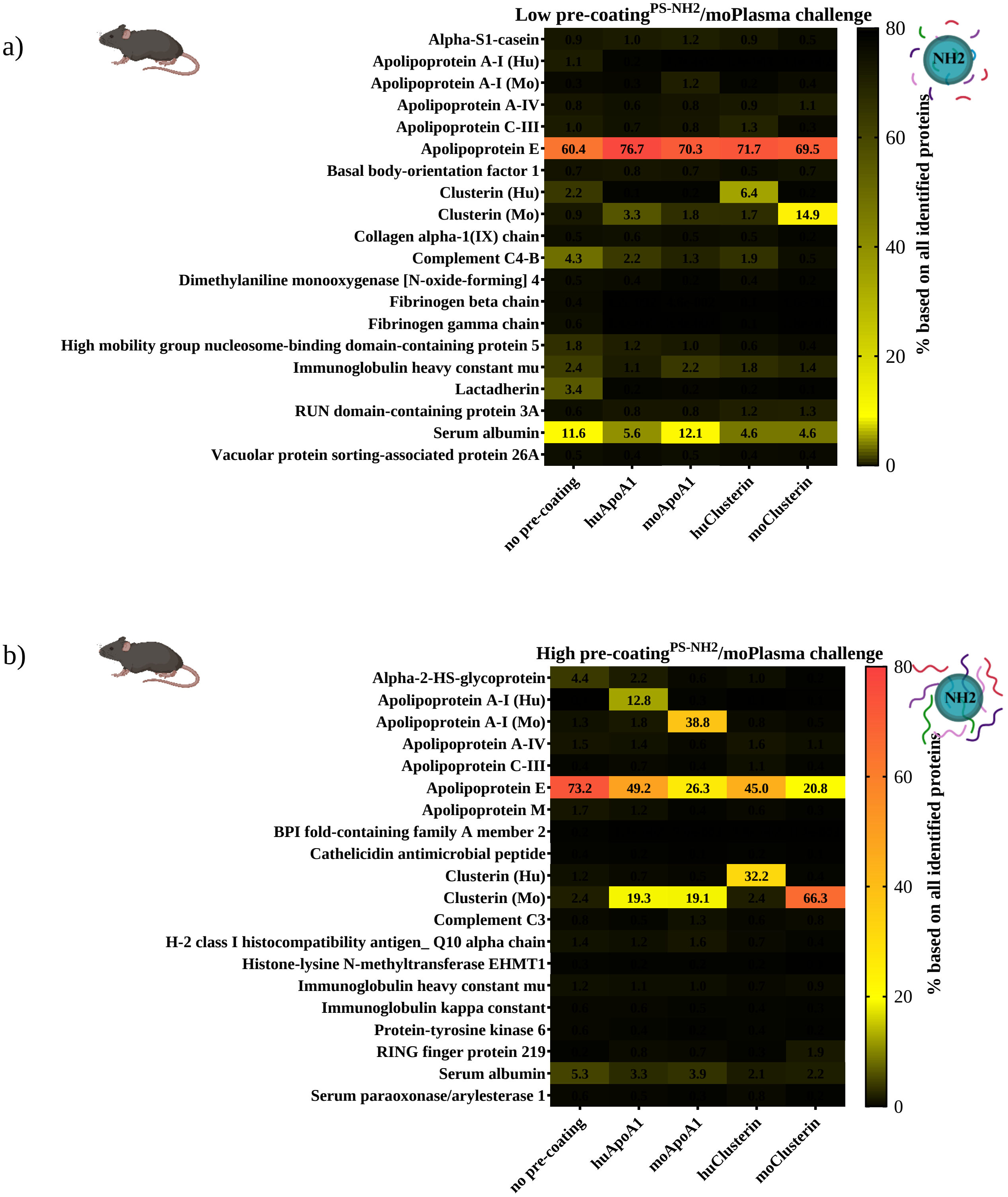

Figure S14. LC-MS analysis of the corona composition: pristine and ApoA1 or clusterin a) Low: 30 µg, b) High: 120 µg, per 0.05 m^2 surface area pre-coated PS-NH2 NPs, and then challenge with mouse plasma.

a)

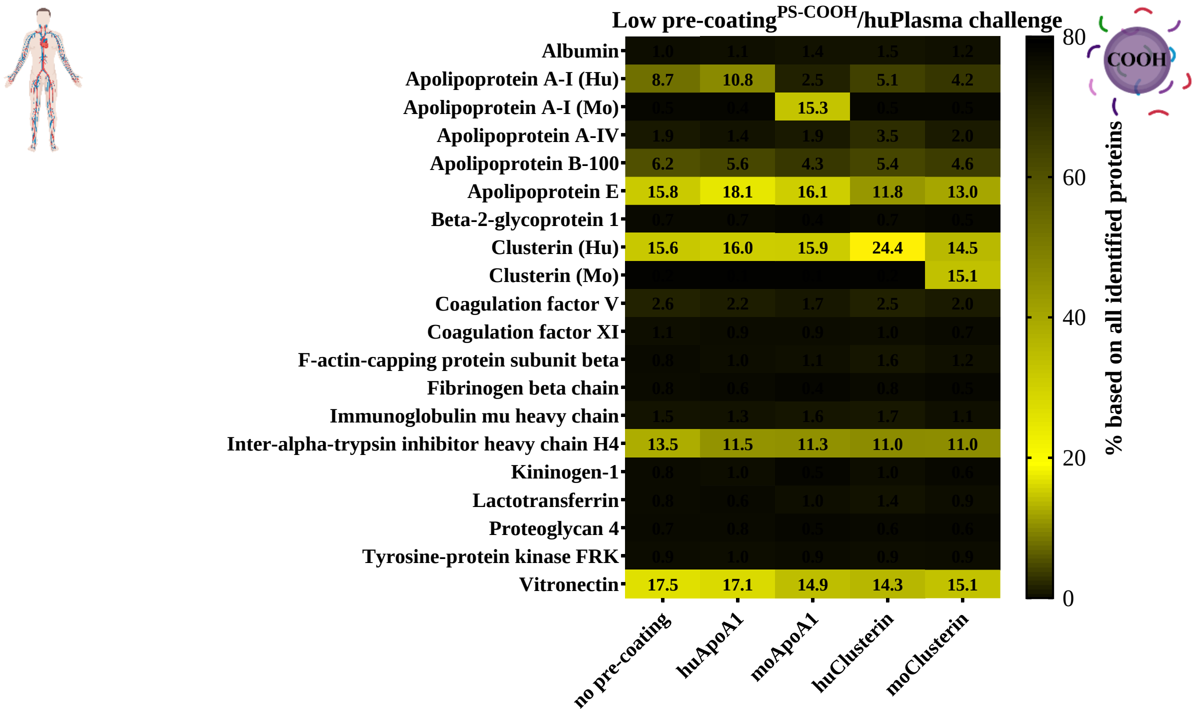

b)

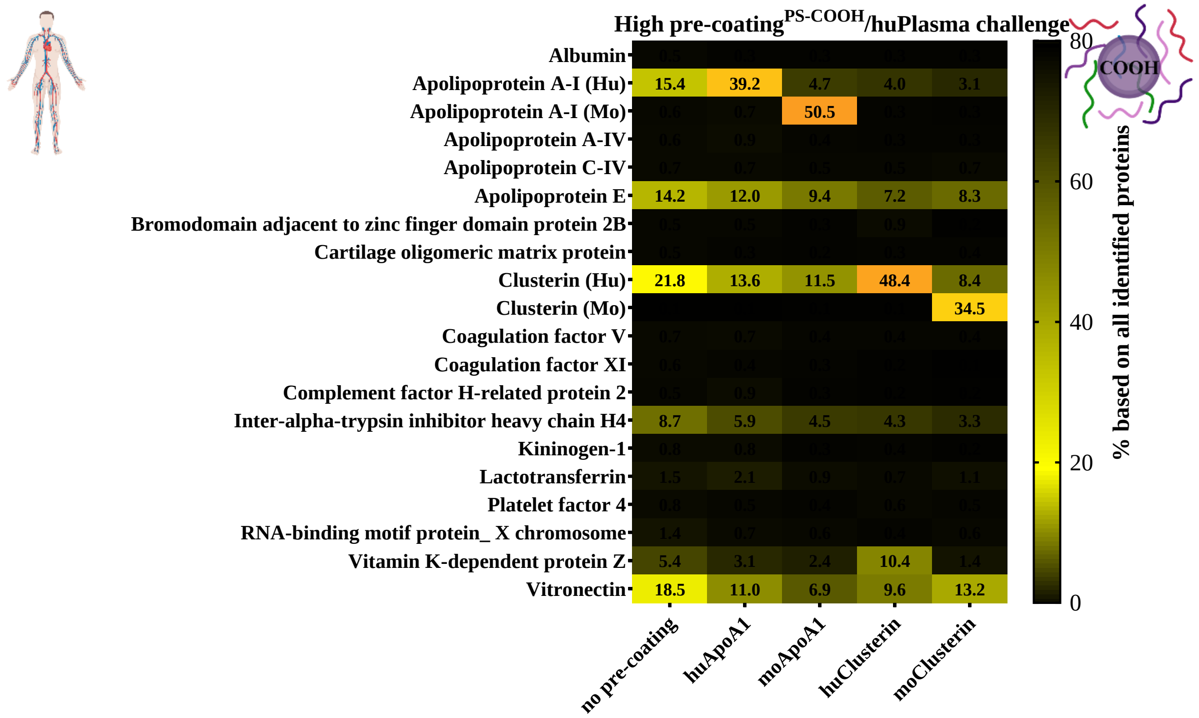

Figure S15. LC-MS analysis of the corona composition: pristine and ApoA1 or clusterin a) Low: 30 µg, b) High: 120 µg, per 0.05 m^2 surface area pre-coated PS-COOH NPs, and then challenge with human plasma.

a)

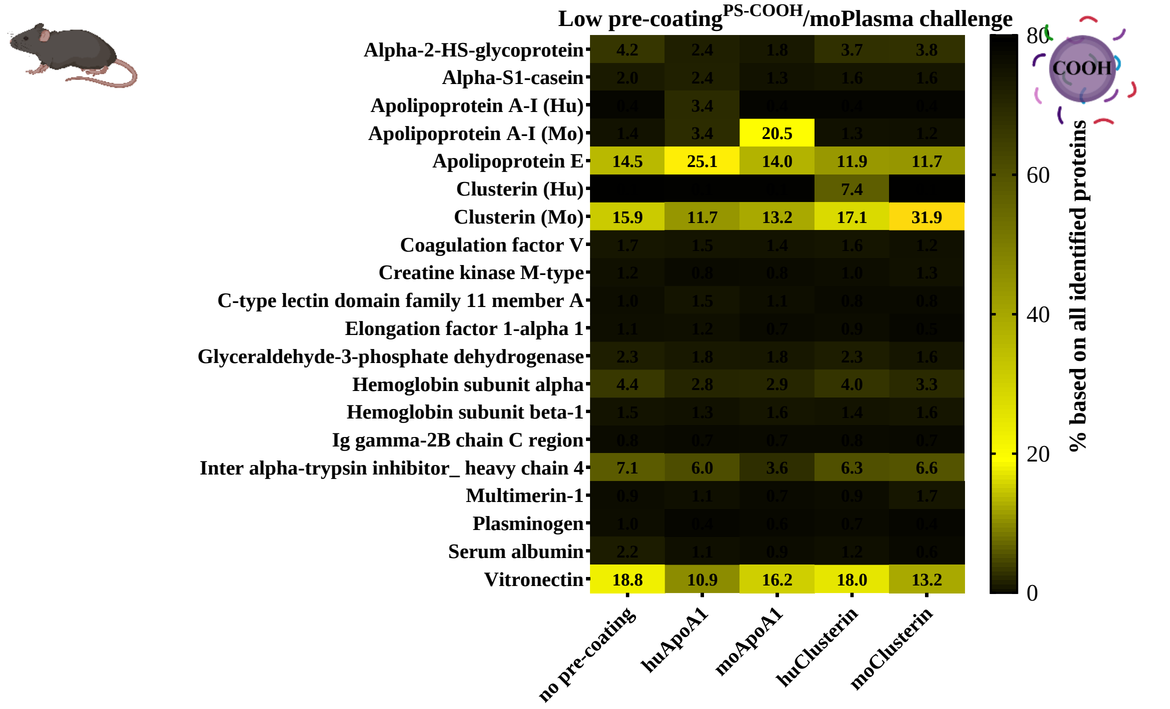

b)

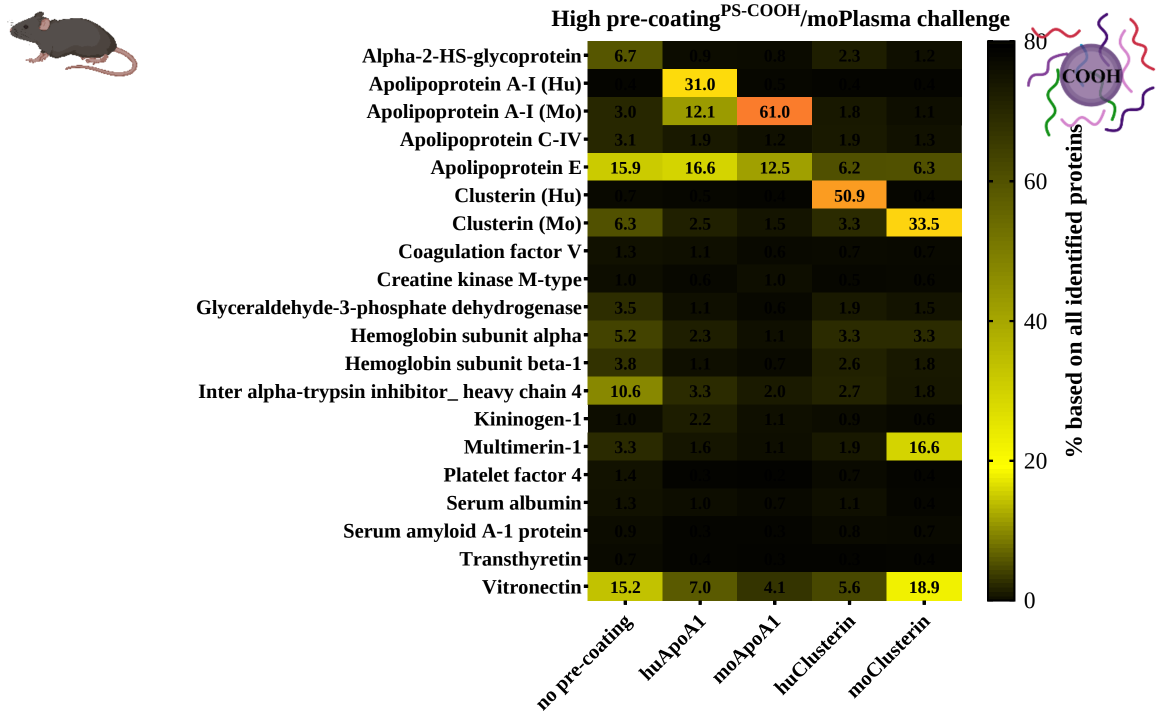

Figure S16. LC-MS analysis of the corona composition: pristine and ApoA1 or clusterin a) Low: 30 µg, b) High: 120 µg, per 0.05 m^2 surface area pre-coated PS-COOH NPs, and then challenge with mouse plasma.

a)

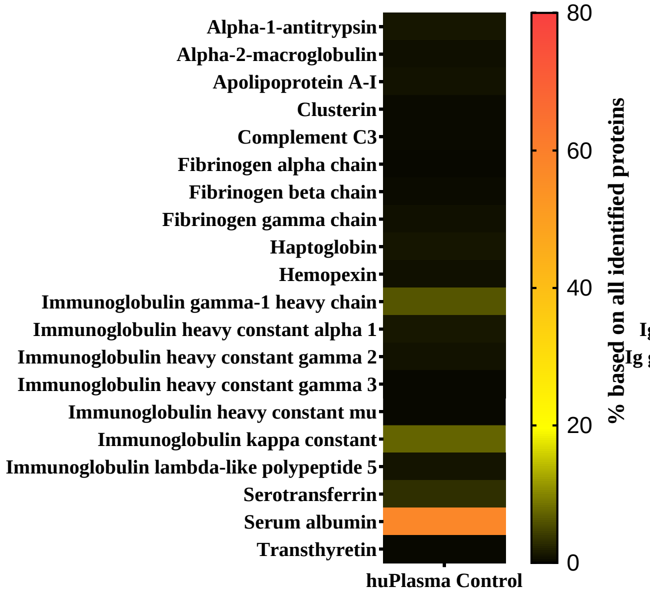

b)

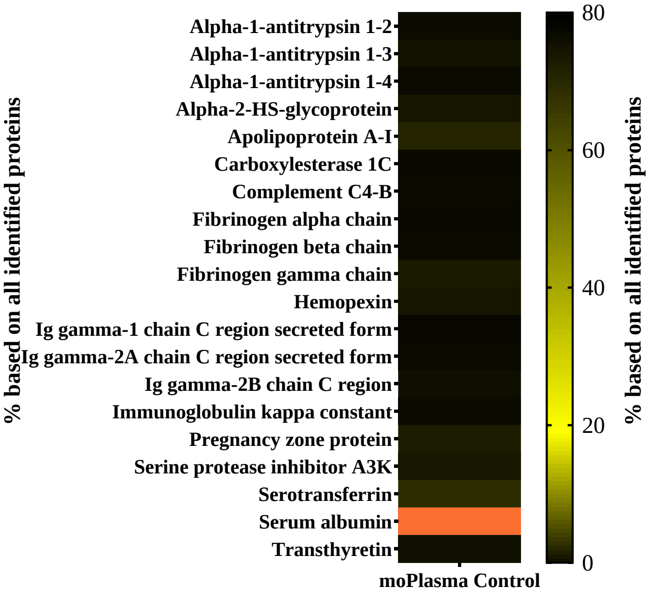

Figure S17. LCMS protein identification of a) human plasma and b) mouse plasma control. Values are expressed as mean ± SD triplicates

a)

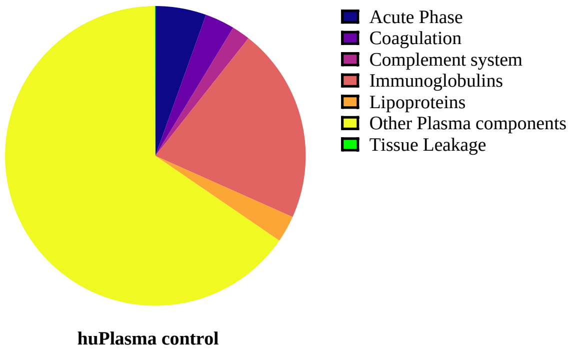

b)

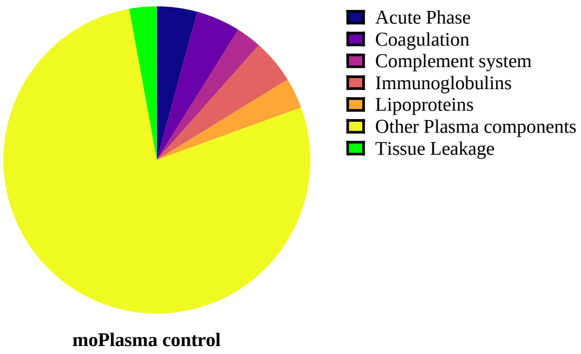

Figure S18. LCMS protein classification of a) human plasma and b) mouse plasma control.

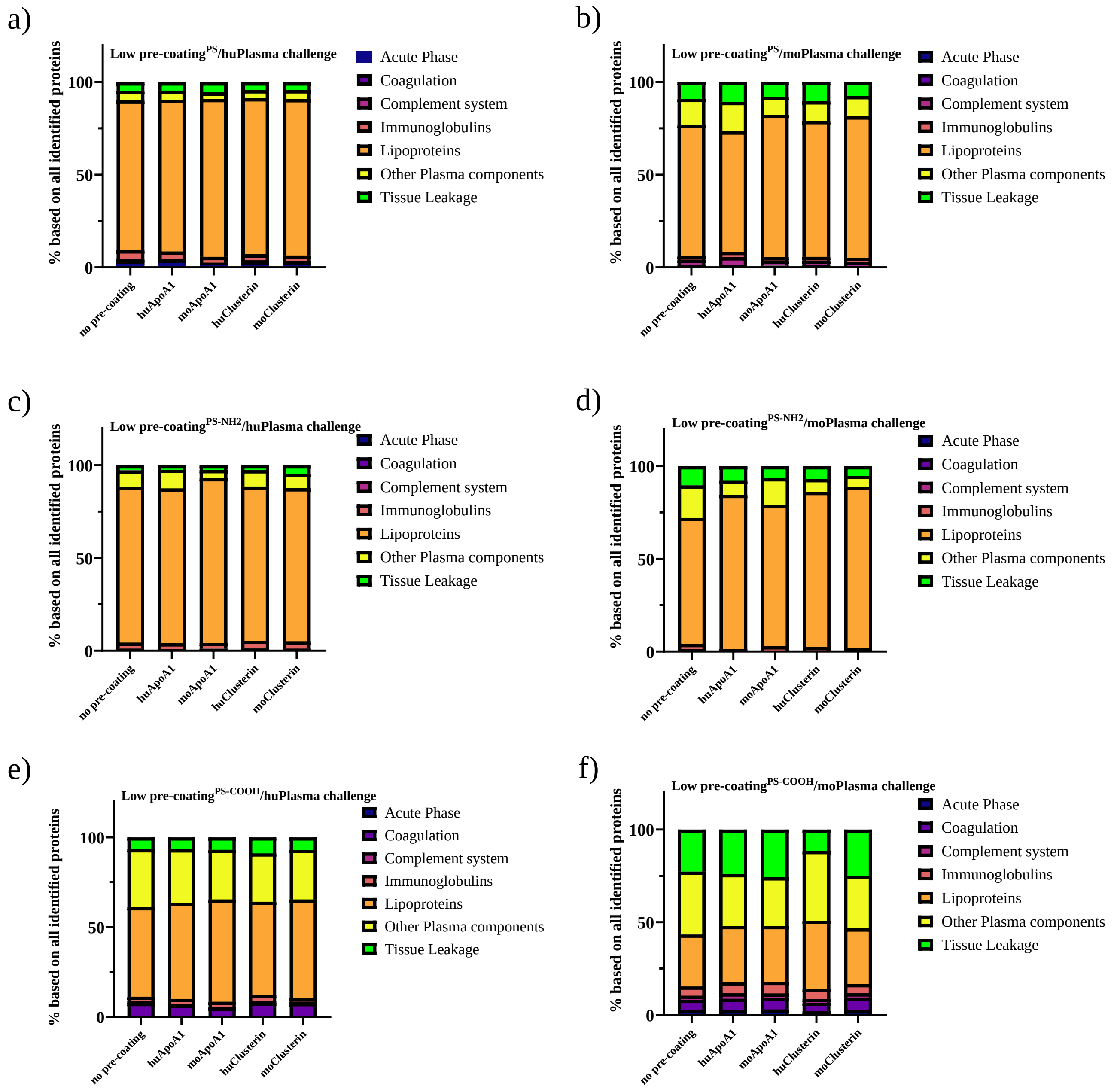

Figure S19. LC-MS Analysis: Protein classification of hard corona proteins: Pristine and low amounts pre-coated (30 µg per 0.05 m^2 surface area) a-b) PS, c-d) PS-NH2, and e-f) PS-COOH NPs, and challenge with human (left) or mouse (right) plasma. Proteins were classified based on their biological function. Values are expressed in % based on the total amount of all identified proteins.

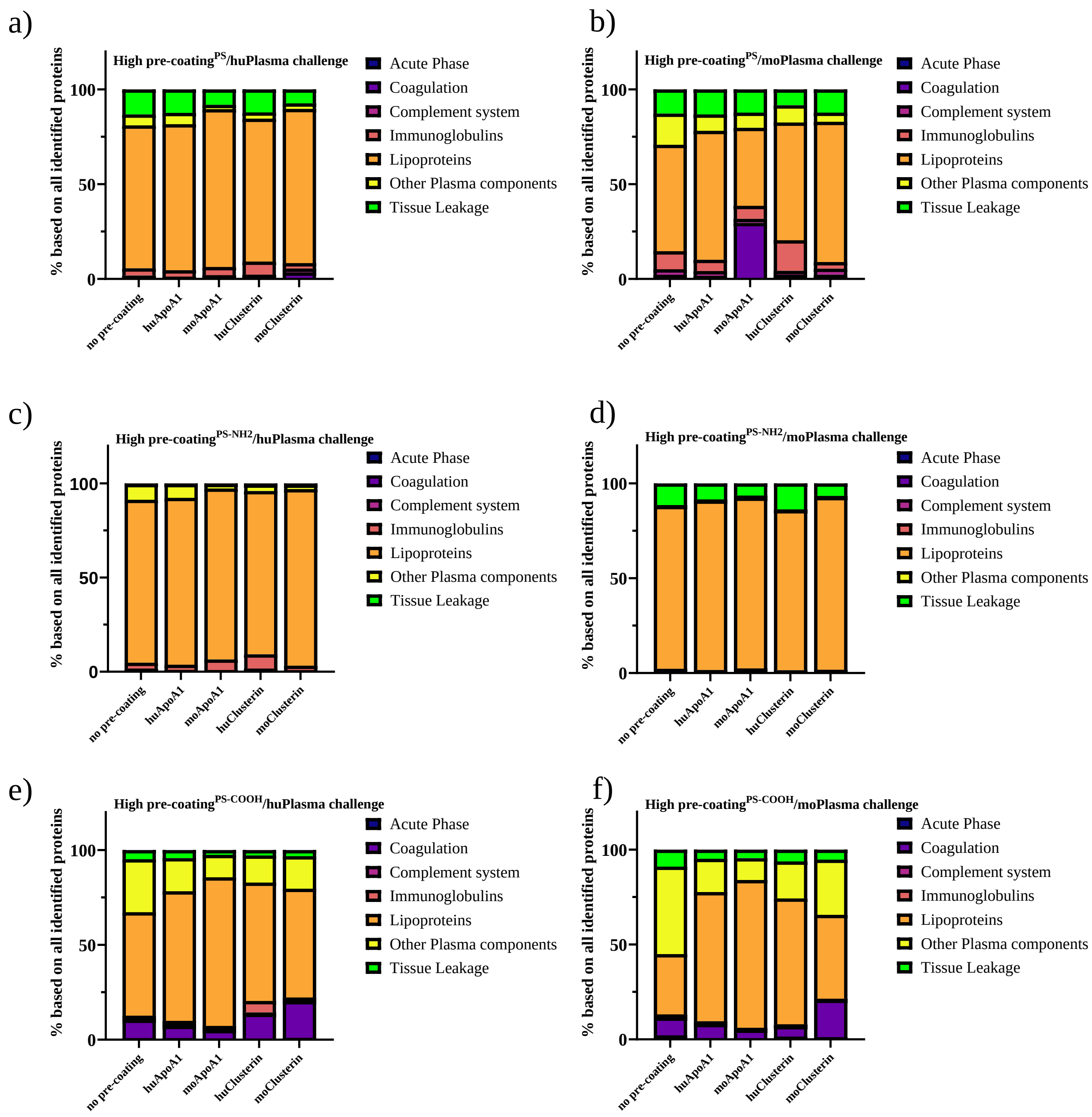

Figure S20. LC-MS Analysis: Protein classification of hard corona proteins: Pristine and high amounts pre-coated (120 µg per 0.05 m^2 surface area) a-b) PS, c-d) PS-NH2, and e-f) PS-COOH NPs, and challenge with human (left) or mouse (right) plasma. Proteins were classified based on their biological function. Values are expressed in % based on the total amount of all identified proteins.
